# Navigating identity and migration: The lived experiences of Indian immigrant women in the Netherlands

**DOI:** 10.1177/17455057261479596

**Published:** 2026-08-14

**Authors:** Pankhuri, Sarah Jay, Gulnaz Anjum

**Affiliations:** 1 Department of Psychology, 8808 University of Limerick, Limerick, Ireland; 2 Department of Psychology, University of Oslo, Oslo, Norway

**Keywords:** migrant women’s health, identity negotiation, racialised institutional norms, emotional simultaneity, moral economies of care, bounded hybridity, interpretative phenomenological analysis (IPA)

## Abstract

**Background:**

Highly skilled migrant women are often framed as beneficiaries of global talent mobility, yet their psychosocial realities remain poorly understood. Indian women navigate racialised institutional expectations, gendered cultural norms, and emotional labour that directly affect wellbeing. Existing integration models overlook how power, belonging and misrecognition shape women’s mental health in everyday life.

**Objectives:**

This study examines how highly skilled Indian immigrant women in the Netherlands negotiate cultural, emotional and professional identities; how they experience racialised institutional norms; and what strategies they use to sustain wellbeing within unequal social and workplace environments.

**Design:**

A qualitative study using Interpretative Phenomenological Analysis (IPA).

**Methods:**

Using Interpretative Phenomenological Analysis (IPA), we conducted semi-structured interviews with 12 highly skilled Indian women aged 24 to 49 years (residing 2–8 years). Analysis followed idiographic, reflexive procedures, examining affective, cultural and identity-based meaning-making with attention to context and embodied experience.

**Results:**

We developed four themes: (1) Internalization of inherited moral economies of care and humility; (2) Encounters with Dutch Norms that impose assertiveness and emotional restraint as markers of competence; (3) Identity Negotiation through selective unlearning, cultural anchoring and strategic self-presentation; and (4) Ambiguous Selves, defined by ongoing emotional vigilance and partial belonging. These findings culminate in the concept of Emotional Simultaneity, the continuous coexistence of conflicting emotional and cultural demands.

**Conclusion:**

Highly skilled Indian women’s wellbeing is an ongoing, relational process shaped by structural power and emotional negotiation. Emotional Simultaneity offers a new lens for understanding migrant women’s mental health and highlights the need for inclusive, context-sensitive policies that recognise emotion as a determinant of health.

## Plain Language Summary

Many Indian women move to the Netherlands for skilled jobs in fields such as technology, research, and business. They are often described as success stories of global migration. Much less is known about how this move affects their emotional lives and mental health. We wanted to understand what daily life feels like for these women. How do they manage the expectations of their Indian upbringing alongside the expectations of Dutch society and workplaces? What do they do to protect their wellbeing? We interviewed 12 highly skilled Indian women living in the Netherlands. They were between 24 and 49 years old and had lived in the country for two to eight years. We used a research method designed to explore personal experience in depth, paying close attention to how each woman made sense of her own life. Four patterns stood out. First, the women carried values from their upbringing that emphasised care for others and humility. Second, they faced Dutch workplace norms that treated directness and emotional restraint as signs of competence. These two sets of expectations often clashed. Third, the women managed this clash in creative ways. They let go of some old habits, held on to cultural anchors that mattered to them, and adjusted how they presented themselves in different settings. Fourth, many described feeling watchful and never fully at home, in either culture. We call this experience Emotional Simultaneity. It means living with two conflicting sets of emotional and cultural demands at the same time, every day. This constant balancing act takes real effort and can wear on mental health. Our findings suggest that employers and policymakers should treat emotional experience as part of health. Support for migrant women should recognise the invisible work of living between cultures.

## Introduction

Global frameworks such as the Sustainable Development Goals (SDGs) have provided an unprecedented architecture for measuring progress through seventeen goals, 169 targets, and 232 indicators. Two goals speak most directly to this study: SDG 3 (good health and well-being) and SDG 5 (gender equality).^1^ Yet as 2030 nears, these metrics miss the relational and psychological dimensions of wellbeing that shape how people endure change. Migration exposes this omission most vividly: through the movement of people, the contradictions between structural progress and lived experience become visible where material opportunity coexists with affective uncertainty. Women’s trajectories reveal these contradictions most acutely: they sustain the social and affective infrastructures that make transnational life possible, even as they bear disproportionate burdens of adaptation and recognition.

This study explores these complexities through the experiences of highly skilled Indian women in the Netherlands, positioned at the intersection of privilege and precarity. Their migration pathways, often celebrated as global success stories, conceal subtler psychological challenges. Navigating unfamiliar gender and workplace norms, they balance competing expectations of belonging and competence. Their mobility brings material stability yet exposes misrecognition that erodes everyday psychological well-being and emotional coherence. We read these dynamics through intersectionality, which examines how gender, race, and class operate together rather than separately,^2^ and through theories of recognition and misrecognition, which treat social standing as a condition for psychological integrity.^3^ Belonging is understood as a political and emotional process rather than a fixed status.^4^

Since 2004, the Netherlands’ high-skilled migrant visa has attracted thousands of applicants, with Indian nationals comprising nearly one-third.^5,6^ Globally, Indian migrants form the largest tertiary-educated group across OECD countries,^7^ and the number of highly educated female migrants grew by 80% between 2000 and 2011.^8^ Despite their visibility in policy narratives of talent and integration, the psychological costs of their identity and emotional negotiations remain understudied. Feminist and intersectional scholarship has revealed how gender, race, and class shape migrant women’s experiences,^9,10^ but few studies examine how internalised moral expectations interact with institutional norms to influence wellbeing among highly skilled Indian women in Europe.

Mainstream approaches treat wellbeing as a post-settlement outcome, measured through employment, language proficiency, or social participation.^11,12^ Feminist and psychosocial perspectives instead frame it as an ongoing emotional and relational process: a form of mental health maintenance that unfolds through everyday negotiations of identity, recognition, and belonging.^13,14^ Building on this shift, the present study treats mental health as embedded within these relational and cultural processes: wellbeing is not an endpoint but the very emotional field through which women sustain coherence within unequal systems of power.

Accordingly, this study asks:

(Q1) How do Indian immigrant women perceive their gender, ethnicity, and professional identities within a bicultural context?
(Q2) What challenges and opportunities arise as they negotiate cultural expectations and professional roles?
(Q3) How do they sustain coherence and wellbeing across workplace and personal domains?

Together, these questions reposition well-being and, by extension, mental health as an ongoing emotional negotiation of belonging within unequal power structures. They frame adaptation not as assimilation but as the psychological work of sustaining coherence, dignity, and resilience across intersecting social domains.

We argue that the wellbeing of highly skilled Indian migrant women is sustained through Emotional Simultaneity: the continuous affective labour of holding conflicting cultural and professional commitments that migration leaves unresolved. This study makes three contributions. Conceptually, it introduces Emotional Simultaneity as a lens for understanding migrant wellbeing. Empirically, it documents the psychosocial realities of highly skilled Indian women in the Netherlands, a group that policy narratives celebrate but research has overlooked. Theoretically, it reframes wellbeing as a relational, processual, and emotionally negotiated achievement rather than a post-settlement outcome.

## Theoretical framework

This study draws on four bodies of theory to explain how identity negotiation unfolds within historically embedded power relations. Postcolonial and intersectional theory situate the macro-level power fields that shape recognition and belonging.^2,15^ Ecological and internalisation models trace the micro-level moral development that precedes migration.^16,17^ Acculturation and hybridity theory analyse the meso-level identity practices that migrants enact within host-country institutions.^18,19^ Affect theory supplies the emotional dimension, showing how adaptation is sustained through continuous affective work.^20^ We integrate these strands around a central construct, Emotional Simultaneity, which we define below.

At its core is Emotional Simultaneity, which theorizes migrants’ parallel commitments to conflicting cultural norms without full resolution. Rather than choosing between inherited moral economies and host-society expectations, migrants sustain simultaneous attachments that demand ongoing emotional labour across institutional and relational obligations. This emotional work underpins psychological coherence and fatigue alike, linking identity negotiation to the everyday texture of wellbeing.

### Structural foundations: Postcolonial and intersectional power fields across contexts

Dominant models of cultural adaptation^18^ conceptualize migration as individual adjustment between heritage and host culture. While influential, they overlook how gendered, racialized, and classed hierarchies shape whose identities and emotions are recognized.^15,21^ Even ecological extensions^22^ under-theorize the systemic hierarchies that constrain adaptive possibilities and generate psychosocial stress for migrant women.

Postcolonial and intersectional analyses re-situate migration within transnational structures of inequality.^10^ In the Netherlands, neoliberal and postcolonial legacies converge within a cultural logic of institutional whiteness, where meritocracy and autonomy are racialized markers of competence.^23,24^ In South Asia, colonial and patriarchal moral economies valorize care, modesty, and duty as feminine virtues.^25–27^ When these norms meet Dutch expectations of assertive autonomy, women must continually regulate emotion and self-presentation to sustain legitimacy. Migration thus exposes how power structures generate psychosocial stress and shape the emotional negotiation of identity, agency and wellbeing.

### Internalization processes: Ecological development and moral economy transmission

While postcolonial and intersectional theories clarify social positioning, psychological accounts explain how structures become self-regulatory emotional frameworks. Migrants carry moral economies that appear self-authored while reflecting structurally constrained agency.

The Model of Internalisation of Normative Social Harmdoing (MINSOH)^17^ describes how collective norms fulfill needs for competence and relatedness, yet neglects how power restricts which norms are available. Embedding MINSOH within Bronfenbrenner’s Ecological Systems Theory^16^ situates this process within nested contexts: families transmit gendered expectations of care and relational virtue.^28^ Mesosystem contexts, schools, religious institutions, and peer networks, reinforce these emotion norms.^29^ Exosystem influences, such as community surveillance and diasporic ties, amplify conformity pressures.^30^ At the macrosystem level, patriarchal and postcolonial ideologies define virtue itself, framing autonomy as relational responsibility rather than individual freedom.^27,31^ Through this multilayered scaffolding, structural power becomes internalised virtue, producing emotionally invested identities that migrants carry into new institutional settings.

### Institutional confrontation: Navigating post-migration norms

After migration, inherited moral economies encounter institutions structured by neoliberal productivity demands, gendered state discourses, and racialized professionalism.^26,32^ Highly skilled Indian women often face workplace expectations of assertiveness and self-promotion that conflict with internalised values and communal obligation.^27,28^ These tensions are interpreted through racialized and gendered lenses, framing restraint as emotional immaturity or insufficient integration.^9,33^ Gender-equality policies further promote an individualized ideal of emancipation, recasting relational duty as dependence.^26^ Such encounters produce structural incoherence,^34,35^ where legal inclusion coexists with social exclusion and belonging remains conditional.

### Third space identity negotiation: Hybridity under constraint

Faced with these tensions, highly skilled migrants neither fully assimilate nor resist. Instead, they engage in Third Space negotiation,^19^ selectively integrating host and origin norms. Rather than blending identities, this entails strategic reperformance,^33^ recalibrating relationality and boundary-setting, based on contextual stakes.^36^

However, hybridity remains constrained: only behaviours legible within racialized, neoliberal norms are recognized as competence.^32^ This continual negotiation enables functionality but often produces fatigue and ambivalence.

### Emotional simultaneity: The affective labour of unresolved contradictions

While Third Space negotiation enables behavioural flexibility, it seldom resolves emotional dissonance. This study introduces Emotional Simultaneity to describe the continual affective labour through which highly skilled Indian women manage dual commitments to relational duty and professional autonomy, modesty and ambition. We define Emotional Simultaneity as the sustained and unresolved coexistence of conflicting emotional and cultural commitments that a person regulates in parallel rather than in sequence. It differs from emotional labour, which describes the management of feeling to meet a single set of display rules.^37^ Emotional Simultaneity describes the parallel management of two incompatible sets of affective and moral demands at the same time. These allegiances coexist rather than unfold sequentially, demanding constant emotional regulation to sustain coherence and belonging. This reflects earlier work on “life in limbo,” which shows how prolonged uncertainty, suspended identities and constrained agency generate chronic emotional strain among displaced and migrant populations.^38^ Such limbo states underscore that psychological adaptation is not a linear process, but an ongoing negotiation shaped by structural insecurity and moral commitments.

Affect Theory clarifies this process by viewing emotions as social forces circulating through hierarchies that define which feelings are legitimate.^20^ Adaptation therefore involves navigating affective regimes of recognition as much as behavioural ones. Emotional Simultaneity extends acculturation and hybridity theories, typically focused on cognitive or behavioural adjustment, by showing how emotional processes sustain adaptation under constraint. It also refines internalization models such as MINSOH by revealing how structural hierarchies determine which emotional commitments persist after migration. Affective labour functions as both resilience and strain, enabling participation while producing fatigue, anxiety, and partial wellbeing.

### Integrative process model of power-saturated identity negotiation

**Figure 1.**
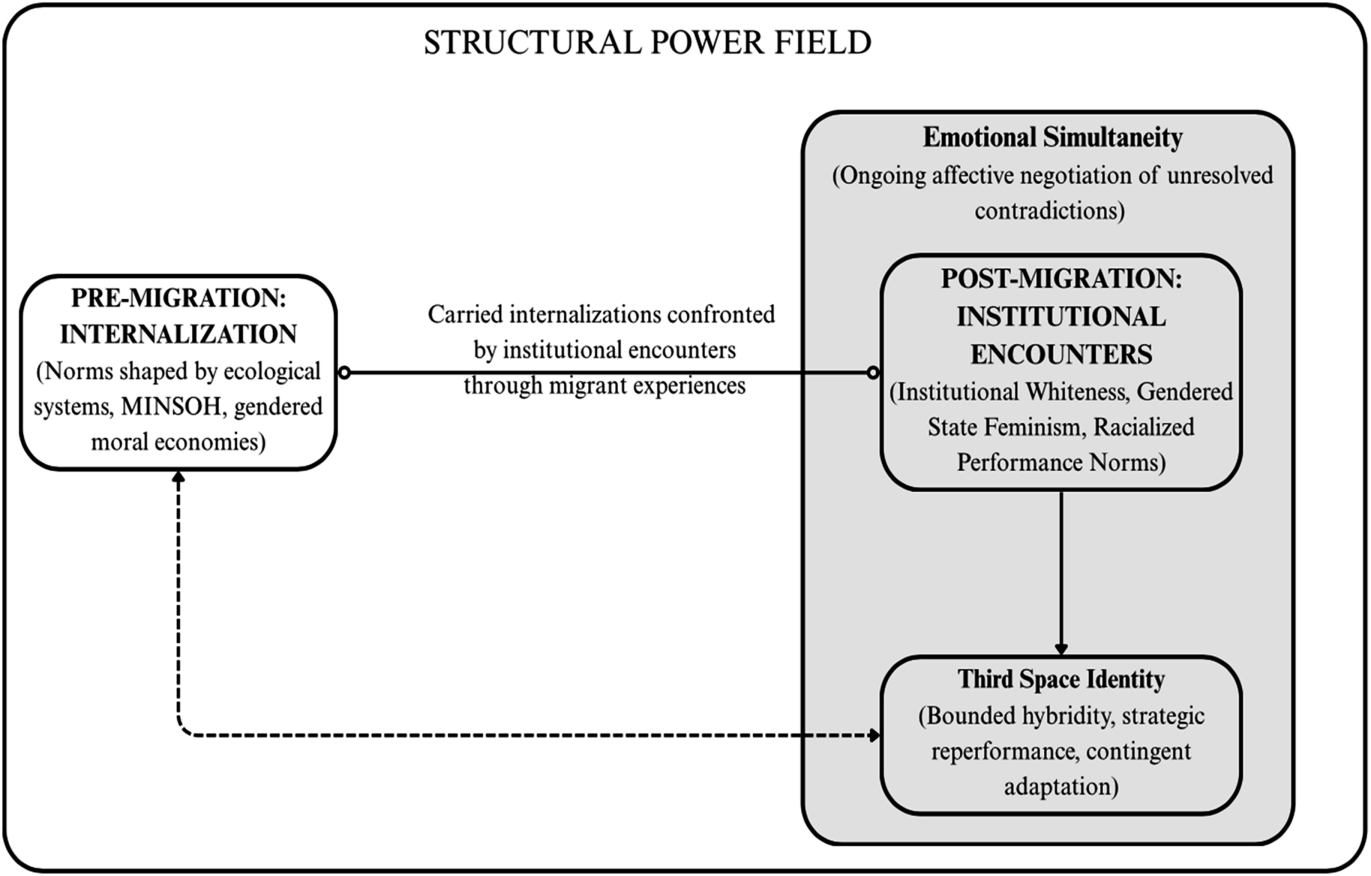
Conceptual model of migrant identity negotiation within structural power fields. *Note.* Internalised pre-migration norms interact with post-migration institutions, producing emotional simultaneity and shaping third space identity formation. Structural power operates throughout as both context and constraint.

As presented in Figure 1, this model conceptualizes migrant identity negotiation as a dynamic psychosocial process unfolding across interconnected structural, cultural, and emotional layers. Migrant identity negotiation thus unfolds across interconnected structural, cultural, and emotional layers: pre-migration socialization creates emotionally charged moral identities; institutional encounters test and transform them; this confrontation activates selective recalibration of behaviours across professional, institutional, and private domains. Overlaying this behavioural negotiation is Emotional Simultaneity, the simultaneous, unresolved affective attachment to multiple, often contradictory moral frameworks. Although migrants may recalibrate earlier norms through reflection, such reinterpretation remains bounded by institutional legibility and power hierarchies. Adaptation therefore appears not as linear transformation but as a contingent reworking of self within enduring power fields. In foregrounding the structural and emotional dimensions of identity negotiation, this contribution bridges feminist migration research and psychosocial health studies, offering a culturally grounded framework for analysing and supporting migrant women’s mental health.

## Methodology

### Study design and rationale

This study employed a qualitative, interpretative phenomenological design to explore how Indian immigrant women navigate bicultural identity and gendered expectations in Dutch professional settings. Interpretative Phenomenological Analysis (IPA) was selected for its idiographic commitment to lived experience and meaning-making processes.^39^ Unlike narrative inquiry, which foregrounds storied coherence, or feminist phenomenology, which centers gendered embodiment, IPA enabled in-depth examination of how participants interpret intersectional experiences within socio-institutional contexts. Critical discourse analysis was considered but rejected as the study prioritized subjective meaning over discursive structures.

Grounded in a constructivist-interpretivist epistemology, IPA supported detailed, case-by-case analysis that respected individual nuance and emerging patterns across participants. Husserl’s intentionality^40^ and van Manen’s^41^ perspectives on embodied meaning informed sensitivity to participants’ positioning. IPA’s flexibility allowed exploration of intersecting dimensions of culture, gender, and profession without imposing theoretical frames, keeping emergent themes anchored in participants’ language and realities.

Given the limited scholarship on Indian immigrant women in Europe, IPA offered a context-sensitive and ethically grounded approach that privileged depth and interpretive integrity over generalizability.

### Research ethics

Ethical approval was granted by the Education and Health Sciences Research Ethics Committee, University of Limerick (approval number: 2025_01_19_EHS; approval date: 19 January 2025). Informed consent detailed confidentiality, voluntary participation, and withdrawal rights. Consent was obtained verbally at the start of each interview and audio-recorded, since interviews were conducted remotely. Each participant received the participant information sheet in advance and confirmed consent on the recording before the interview began. Interviews were conducted via Microsoft Teams in private settings to promote psychological safety. A participant-centered approach used open-ended, non-directive prompts with participants free to pause, skip, or redirect conversations. As an act of symbolic agency, participants selected pseudonyms drawn from Indian warrior women, imbuing the process with personal resonance and resistance narratives.

### Participants

The twelve Indian women in this study were aged 24 to 49 years; seven were single and five married. They had lived in the Netherlands for two to eight years, with eight residing for five years or more. Most occupied mid-to senior-level professional roles across consulting, therapy, international relations, sustainability and, predominantly, the technology, engineering and data sectors. This variation allowed us to capture both early and more established migratory experiences. The research questions call for depth rather than breadth, so we sought variation in age, marital status, professional sector, and length of residence rather than demographic representativeness. A sample of twelve participants is consistent with IPA guidelines,^39^ which prioritise small, purposive samples for idiographic depth. While broader qualitative frameworks often reach saturation with 10–12 interviews,^42^ IPA emphasises experiential richness over numerical thresholds, making this sample appropriate for examining complex processes such as bicultural identity negotiation. Data saturation in the conventional sense was not our analytic goal, since IPA prioritises experiential depth over the recurrence of categories. See Table 1 for more details about participants.

**Table 1.** Participant demographics, duration of stay in the Netherlands, and occupation.

Name (pseudonyms)	Age (years)	Residence in Netherlands (Years)	Occupation
*Onake Obavva*	*33*	*3*	*Senior Technical Program Manager - Technology*
*Madam Bhikaji Cama*	*24*	*5*	*International Relations*
*Kamala Devi*	*27*	*5*	*Sustainability Consultant*
*Jhansi Ki Rani*	*49*	*2*	*Senior Technical Program Manager - Technology*
*Savitribai Phule*	*31*	*5*	*Machine Learning Engineer*
*Rani Arantribai Lodhi*	*46*	*7*	*Therapy Practitioner*
*Uda Devi*	*30*	*3*	*International Tax Lawyer*
*Aruna Asaf Ali*	*31*	*6*	*Product Manager Data*
*Durgabai Deshmukh*	*31*	*2*	*Strategy Consultant*
*Belawadi Mallamma*	*26*	*8*	*Project Engineer*
*Rani Lakshmi Bai*	*29*	*6*	*Process Integration Engineer*
*Jhalkari Bai*	*29*	*6*	*Process Integration Engineer*

### Sampling procedures

Twelve Indian immigrant women were recruited using purposive and snowball sampling. Eligibility criteria included self-identification as an Indian immigrant woman, at least two years’ residence in the Netherlands, and active professional employment. Recruitment began via professional networks, digital communities, and participant referrals. No participant was previously known to the interviewer, and none refused to take part or withdrew after consenting. Variation in age, professional sector, and migration duration ensured both convergent and divergent identity trajectories. Purposive and snowball sampling carries a risk of network bias, since referrals tend to reproduce the social worlds of the initial participants. We worked against this by recruiting across several professional sectors and digital communities. We still acknowledge that the sample reflects women with the resources and confidence to take part.

### Data collection

Twelve semi-structured interviews (60 to 90 minutes each) explored cultural belonging, workplace negotiation, and personal transformation. Data was collected between February 2025 and March 2025. Demographic information (e.g., age, migration history) emerged organically during conversation. Sample prompts included “What does ‘belonging’ look like to you in each context?” and “In what ways do your family’s cultural traditions or values shape your understanding of gender and ethnicity?”. All interviews were conducted, audio-recorded and transcribed verbatim by the first author, preserving pauses and affective cues to preserve experiential nuance. By affective cues we mean audible features of speech that carry emotional meaning, including pauses, changes in pace, laughter, and shifts in tone. Each participant took part in one interview; no repeat interviews were conducted, and transcripts were not returned to participants for comment or correction. The full semi-structured interview guide is provided in Supplementary Material (Interview Guide). The guide was developed by the research team for this study and was not a standardised or previously validated instrument. The reporting of this study conforms to the Consolidated Criteria for Reporting Qualitative Research (COREQ).^43^ The completed checklist is provided as Supplementary Material.

### Data analysis

Analysis followed IPA procedures.^39^ Transcripts were read multiple times and annotated for descriptive, linguistic, and conceptual meaning. Idiographic analysis preceded cautious cross-case synthesis, consistent with IPA’s double hermeneutic: participants making sense of their experiences while the researcher makes sense of their sense-making.

“Superordinate domains” (culture, gender, professional) were generated inductively. By superordinate domains we mean the broad experiential areas that organised the analysis, and by meaning structures we mean the recurring ways participants made sense of experience within each area. Within each, meaning structures were identified before recognizing shared patterns; divergence was preserved to maintain complexity. Multiple coding cycles, memoing, and supervisory consultation ensured analytic rigor. Coding was carried out manually, without the use of qualitative data analysis software. Participants did not provide feedback on the findings. Reflexive memos tracked evolving interpretations and distinguished participant meaning from researcher projection. Superordinate themes such as boundary work, strategic identity performance, and affective states emerged inductively from participants’ own words and metaphors. Linguistic features, metaphor, emotion, and silences, were treated as rich data rather than analytic noise, supporting a layered psychosocial interpretation. A detailed audit trail of the analytic procedure, including the coding workflow, idiographic domain tables, and cross-case integration steps, is available in Supplementary File 2 (Analytic Process). The summary table of final themes and subthemes are available in Supplementary Material.

### Reflexivity and positionality

All authors who contributed to this paper identify as females. While the first authors’ shared ethnic and gendered positionality fostered cultural fluency, it also risked assumed understanding, participant over-alignment, or projection of shared narratives. GA, the third author herself was an immigrant from South Asia in Ireland at the time of this study. This offers an informed insider perspective on the experiences of migrant women navigating life as perceived outsiders. A shared cultural background enabled deep engagement with participants’ narratives and a nuanced understanding of cultural references. These dynamics were critically engaged throughout data collection and analysis via reflexive journaling, memoing, and supervisory dialogue.

Clarifying prompts and memoing bracketed assumptions challenged over-accommodation or researcher assumption, treating subjectivity as an analytic resource. Attention to power asymmetries, especially where resistance or silence signaled contested meaning-making, ensured ethically grounded interpretation that honored both individual narratives and shared structural conditions. A full reflexivity statement outlining the researcher’s positionality and interpretive stance is included in Supplementary Material.

## Results

This analysis traces how highly skilled Indian immigrant women in the Netherlands navigate identity transition, from internalised Indian moral and gendered frameworks to Dutch cultural norms. Rather than linear assimilation, the process reflects continuous negotiation between inherited scripts, host expectations, and evolving self-understandings.

We developed four interconnected themes through our interpretative engagement with the data. We use the word developed rather than emerged to acknowledge our active role in shaping the analysis. Internalization (Theme 1): Early gendered and cultural expectations in India became moral obligations of duty, modesty, and care, shaping professional and personal identity; Encounter with Dutch Norms (Theme 2): Migration confronted these internalised scripts with Dutch ideals of assertiveness and autonomy, generating dissonance and conditional belonging; Active Identity Negotiation (Theme 3): Participants engaged in “boundary work,” selectively retaining and redefining norms to construct self-authored cultural hybridity; Ambiguous and Unfinished Selves (Theme 4): Despite adaptation, identity remained unsettled, transformed but constrained by systemic gatekeeping and emotional simultaneity.

Together, these themes represent psychological, cultural, and structural layers of negotiating selfhood in the Dutch context. Below, each theme is presented as a continuous narrative built around the most illustrative quotations, read through the study’s theoretical lenses.

### Theme 1: “I was not raised to be independent. I was really raised to be a daughter”

Participants described learning gendered, cultural, and professional expectations through early emotional attunement and everyday routine. Duty, modesty, and care were internalised as self-regulation, offering belonging but limiting autonomy. These early habits formed enduring patterns for managing emotion, obligation, and self-worth.

The central point of this theme is that gendered and cultural virtue was learned early and then carried forward as self-regulation. Read through an intersectional lens, femininity was taught as emotional caregiving. Savitribai recalled that her parents “would feel comfortable sharing their emotional problems with me… like I might have a better response than the men in their life.” The role built relational skill and trained self-silencing at the same time. Modesty carried the same lesson. Jhalkari said she “never had the chance to dress for [herself]” and had to dress so as to “never attract the other gender.” The external gaze became an internal monitor. Cultural virtue extended this discipline into the collective. Diligence was moralised, as one participant put it: “Hard working was one of the things like no matter what. Yeah, just work hard and things will work out.” The fear of “log kya kahenge”, what people will say, tied worth to approval. These habits had a cost. One participant linked relentless work to her health: “I had severe vitamin D deficiency… I had an acute depression episode.” Across accounts the pattern holds. Belonging was offered in exchange for emotional restraint.

#### Sub theme 1.1: “They always wanted you to be soft…to be the good girl who never showed skin”

Femininity was learned through everyday interactions, where modesty, care, and deference signalled virtue. These values, absorbed early through family routines and emotional labour, shaped identity as self-regulation. Participants were expected to manage others’ emotions, equating calmness and empathy with goodness. Emotional labour thus became moral expectation rather than choice.

These lessons were lived as ordinary family life. Jhalkari recalled, “I never really saw much of a gender difference inside my house… Of course, there were obvious differences. My dad got the money for the family, and my mom stayed at home… but there were just two people doing stuff.” Durgabai described the sanction that met any departure from the script: “Even my husband gets treated differently because he is an equal partner… they frown at me for enabling him.” The norm felt natural while it was obeyed and visible only when broken.

Femininity centered on modesty as a moral code and safety mechanism. The female body became a site of vigilance governed by anticipated scrutiny, where self-presentation was adjusted to preserve respectability. Some described their families as egalitarian, their narratives revealed deeper ambivalence. Role differences were often explained as practical, fathers earning income, mothers managing home, thereby naturalising gender divisions that later felt constraining.

Participants also internalised these constraints in decision-making, rationalising choices male siblings accessed by default and cultivating anticipatory self-censorship. These internalisations extended into adulthood, where deviation from feminine self-sacrifice invited subtle judgment. Equality was often perceived as failure to perform expected service and restraint. Gendered virtue created emotional security through predictability and moral clarity, yet confined autonomy within compliance. Calmness, modesty, and deference offered safety but required continuous emotional control, turning restraint into both strength and quiet strain.

#### Subtheme 1.2: “They would always tell us to be humble and modest… not attach yourself too much to how much money you have”

If gendered upbringing taught restraint within the family, culture extended that restraint into the collective gaze. Cultural identity was shaped by moral teachings, class aspirations, communal surveillance. Humility, restraint, and diligence anchored self-worth, linking morality to endurance rather than outcome.

Cultural worth was measured against a Western standard. One participant noticed the hierarchy plainly: “A lot of people would, like, downplay Indian-ness…in favour of Western thing… in a very matter-of-fact we’re-below-you type of way.” The same women found relief in cultural ritual: “The minute I’m eating home-cooked food… or getting bombarded by all of my old cultural references… it feels like home.” Belonging and inferiority sat together in the same cultural frame.

The omnipresent *log kya kahenge* (“what will people say”) gaze turned social regulation, into self-monitoring, tying respectability to conformity. Women learned to anticipate evaluation, balancing pride in tradition and pursuing individuality. This constant vigilance turned comparison into habit: comparison became a measure of moral worth and safety.

Postcolonial class dynamics deepened this tension. Western markers of taste and achievement were equated with progress, while overt Indianness risked seeming inferior. Such hierarchies produced ambivalence: cultural heritage was a source of comfort, yet also of internalised comparison and quiet shame. Sensory rituals, food, language, festivals, offered emotional safety and collective memory, briefly restoring coherence amid contradictions.

Cultural virtue stabilised identity through shared morality and ritual familiarity but bound self-esteem to external validation. Humility offered moral safety yet sustained chronic self-evaluation and muted pride, embedding ambivalence between collective belonging and individual worth.

#### Subtheme 1.3. “I always paid attention to studies… I do not need to bring my parents down. They’re working… that did not allow me to think of any other extracurricular activity”

The same ethic of composure and humility shaping family and cultural life extended to participants’ approach to work, where diligence became both virtue and validation. Professional identity emerged from early moral socialization anchored in duty, discipline, and scrutiny. Career paths were acts of familial honour rather than self-expression; respectable professions signalled reliability and class stability. Aspiration thus carried the emotional weight of repayment.

Professional ambition was weighed against gendered expectation. One participant described the constraint directly: “My dad still goes back with those beliefs that… being daughters…you should be back before the sunset… now it is my parents, I can convince them… but later after I get married… it will be like wasting my three years.” The same pressure turned into determination: “I knew somewhere deep inside that I have to prove them wrong here. That it is not that I have a family… It is equally important for me.” Ambition and duty were held together rather than resolved.

Anticipated surveillance reinforced this restraint. Decisions were filtered through future respectability: being the daughter who returned home on time or avoided “wasting years” before marriage. Such forward-looking caution cultivated internalised boundaries, narrowing what could be imagined as acceptable ambition.

Work itself was moralised as virtue. Long hours and exhaustion demonstrated seriousness and trustworthiness. Over-performance became a silent defence against doubt that family obligations made women less committed. Success thus required visible endurance: to be tireless was to be beyond reproach. Endurance stabilised self-worth: by working beyond limits, participants preserved moral coherence in systems questioning their legitimacy.

Participants also described moments when the body faltered, fatigue, deficiency, episodes of depression, revealing how duty turned inward as pressure. Moralised diligence anchored identity in responsibility and self-control. It offered coherence, pride, and moral safety, yet confined worth to constant effort, where exhaustion became proof of integrity and rest a failure of character.

#### Synthesis

Together, these subthemes show how gender, culture, and profession formed a single moral landscape of responsibility. Care, humility, and diligence intertwined as moral expectations, securing belonging through self-regulation. Stability was sustained by quiet vigilance: an internalised ethic that transformed endurance into identity. Theme 1 captures the emotional grammar to be dependable, composed, and morally safe, even when it required self-limitation.

### Theme 2: “Back home for someone who’s 29, I’m not doing things a typical Indian 29-year-old would be doing”

This theme examines how participants described clashes between internalised cultural norms and Dutch expectations of directness and individualism. Their accounts reveal tensions between learned values of respect, and relational care and adapting to unfamiliar social and institutional demands. This highlights how inclusion depended on dominant norms, shaping misrecognition and emotional labour in pursuit of belonging.

The central point of this theme is that Dutch norms of directness and autonomy collided with inherited values, and belonging became conditional on adapting. Migration reframed duty itself. One participant described having to unlearn obligation: “Feeling that you need to earn your keep…that was something I needed to get rid of.” Workplace norms drew a sharp line around effort. Another recalled the message from colleagues: “If they find you’re doing something extra… they’d say, get a life. You did your 8 hours.” Read through theories of recognition, only certain ways of working were read as competence. Race sharpened the boundary. One participant named the hierarchy directly: “if you’re digestible brown, you’re fine. But then there’s less digestible brown.” Relationships felt thinner, described as “strictly like business.” The strain was gendered and forward-looking. As one woman put it, “I feel like I deserve to have a good career… and on the other hand, I also kind of want to have a family. So which one do I sacrifice?” The encounter produced misrecognition and a steady emotional labour aimed at belonging.

#### Subtheme 2.1: “I was just taking it quietly… because that’s how you are raised… you should respect your elders… by not talking back to them.”

The emotional discipline established in Theme 1, where restraint and composure signified virtue, met its first rupture in the Dutch context, as participants realised that *the way they had learned to be good no longer made sense here.* What had once been moral strengths now undermined credibility and unsettled the coherence that had guided them.

Respectful restraint, an asset at home, became a liability in Dutch exchanges. One participant noticed the difference in how she was addressed: “They would ask my colleague one way… but they’d ask me the same thing in a different way.” Directness carried a double standard: “If I were to be just as candid as they were to me, they would not be able to take it.” The same candour was read as competence in others and as too much in her.

This conflict was gendered and racialised. The vigilance that once ensured harmony, anticipating others’ comfort and avoiding confrontation, conflicted with ideals of directness. Through racialised lenses, politeness appeared as dependency; the “good-daughter” composure became proof of docility. Virtue now sustained the very stereotypes that undermined them.

Asymmetry shaped everyday communication. When participants mirrored Dutch candour, their tone was judged as confrontational. Communication norms were not neutral but stratified by race and gender. What counted as honesty for some became impropriety for others. The result was a double bind: professionalism in one context violated respectability in another.

Postcolonial hierarchies reinforced this tension, equating improvement with Westernness. The impulse to earn one’s keep reflected both internalised gratitude and external demand to prove worth within Western norms of autonomy. Speaking boldly risked guilt for immodesty; restraint confirmed inferiority.

These accounts show how silence, learned as respect, became a barrier to visibility. The burden of adjustment fell on them, showing inclusion depended on performing dominant communication norms. Emotional habits that once anchored identity now produced anxiety and self-surveillance as participants navigated two incompatible moral codes, obedience as goodness and assertiveness as competence.

#### Subtheme 2.2: “The nightmare that your Indian work life is… that trauma has just come to you in this new country.”

This subtheme examines how participants’ ethic of overperformance, rooted in moral obligation and social survival, was misread within Dutch institutions. Within Dutch workplaces that prized moderation and work–life balance, this diligence appeared excessive, signalling imbalance rather than commitment. What had been moral integrity in Theme 1 now registered as poor cultural fit.

Overperformance became a defence against being found wanting. One participant described her method: “My strategy is only being overly prepared… so that you’re not in a place where you’re just speechless.” A gendered cost ran underneath the effort: “As an organisation… you allow them to take maternity leave… but in reality, you do lose out… because we are bearing the child and he isn’t.” Recognition was available, on conditions that were not equally set.

This misrecognition extended into everyday practice. Overpreparation became self-defence, a way to anticipate judgement, prove competence, and prevent erasure. Yet the effort no longer restored certainty; it drained meaning from their labour. What had been moral control turned into vigilance, sustaining visibility through exhaustion. When colleagues dismissed their intensity as imbalance, the women internalised the hierarchy between balance and endurance, modernity and effort, self-assurance and survival.

This moral inversion evoked deeper insecurities. Exposure to Dutch institutions, efficient yet distant, stirred pride and shame at once: pride in meeting high standards, shame at the suggestion that their background required correction. The emotional cost intensified where gender expectations intersected with policy. Parental and flexibility schemes promised equality yet penalised care, framing motherhood as liability and independence as the only credible professionalism.

Across these layers, moral, emotional, and institutional codes fused into a single contradiction: the harder they worked to belong, the more their labour appeared excessive. Dedication lost its moral value, becoming the very evidence of misfit within systems that rewarded composure over endurance.

#### Subtheme 2.3: “Here generations don’t interact… grandparents are living in elder homes…in India we see ageing around us…we see death around…we see various stages of life around us”

This subtheme examines how the emotional discipline that once organised family and community life met its deepest rupture in the realm of feeling itself. Affectionate dependence, once a sign of loyalty, was recast as need. To remain legible, care had to be translated into the idiom of self-containment.

Family closeness, a source of pride, met incomprehension. One participant kept it private to avoid the same reply: “One very big part of my identity is how close I am with my family… I don’t talk about it a lot because that always ends up with the same question like, ‘Yeah, but so what if it’s your mom and dad?’” Acceptance was also racially graded, as another put it: “if you’re digestible brown, you’re fine. But then there’s less digestible brown.” Belonging was offered within limits set by others.

Relational closeness thus turned into a private burden. To speak lovingly of family risked trivialisation; to maintain distance risked self-erasure. Interactions that appeared professional or efficient to Dutch colleagues felt emotionally hollow to the women, depriving connection of its moral texture. What had once anchored identity now left them socially adrift. This friction deepened along racialised lines. Participants noted that acceptance hinged on being “digestible brown”: emotionally expressive only within limits that kept others comfortable. Those who exceeded these affective boundaries were subtly marked as excessive or foreign. Belonging, therefore, was not only cultural but affective: it required calibrating emotion to dominant sensibilities.

Across these accounts, the challenge was not distance but misrecognition. The emotionality that anchored participants was often illegible in Dutch settings. Belonging depended on performing individualistic emotional norms, leaving them feeling unseen due to normative misalignment. Participants often felt their respectful behaviour was misread as passivity, requiring adaptation to be seen as competent. While many described this pressure to adjust, they also found Dutch people generally welcoming, depending on people’s familiarity with internationals and the broader social context. Belonging was not automatic but negotiated, shaped by both individual adaptation and the awareness of those around them.

### Theme 3: “There are also people who move here who haven’t changed a bit…if you act like someone from back home, then I think there’s no difference between you being here or you being there.”

This theme traces how participants renegotiated identities in a new cultural environment. Through cultural retention, reflection boundary-setting, and strategic self-presentation, they selectively revised inherited norms. These adaptations did not erase past selves but created intentional, emotionally sustainable ways of being in hybrid cultural terrain.

The central point of this theme is that the women actively revised inherited norms through boundary work rather than discarding their past selves. Identity work here was deliberate. One participant held continuity and change together: “We are trying to preserve something and also to reassure our parents that we are OK like we are the same people… we are coping with the current situation.” Independence reframed values from the inside. As one woman reflected, “When I first moved here, I didn’t know what I believed in…your parents instilled certain values…but it takes a while to decide how to make them your own.” Another tied autonomy to a new sense of freedom: “Until you’re independent, you don’t understand what freedom is… no one was expecting you to have that much freedom.” Boundary work meant questioning gendered scripts. One participant wanted “to move out of this traditional role of women in Indian society and try to do my own thing without constantly questioning…Is this the right womanly thing to do?” Read through a hybridity lens, these revisions were selective and self-authored. They built an emotionally sustainable position between two cultural worlds.

#### Subtheme 3.1: “It’s always gonna be India… it’s more about what I associate myself with culturally.”

This subtheme builds on the misalignments described in Theme 2, which left participants suspended between visibility and belonging. Many turned to familiar moral languages of home to restore coherence. Rituals of cooking, language, and hospitality became both care and communication, signalling to families that they remained “the same people” while asserting stability amid host-country uncertainty. These gestures were not nostalgic retreats but adaptive recalibrations: portable moral orders sustaining coherence without demanding conformity

Cultural retention was active maintenance rather than nostalgia. Community and language anchored comfort: “So here we do have a large Indian community… I speak Malayalam that my native language… I feel like that’s my most comfortable place.” Everyday ritual rebuilt a sense of home: “Now I’ve really started cooking my own food and got all my recipes from my grandparents and mother and I’m like enjoying my cooking. I used to miss home and I think now I’m less missing home.” Familiar practice restored continuity in a new place.

By hybridizing Indian and Dutch customs, participants restored meaning to everyday life. Practices like *atithi devo bhava* (“guest as god”), through gestures of greeting and gift-giving, reclaimed dignity in spaces that misread emotion as excess, turning care into quiet resilience. Cultural preservation thus functioned as self-regulated care. Far from resisting integration, it allowed participants to navigate plural belonging with composure and clarity. Through these embodied practices, they reassembled identity not as return to origin but as affective equilibrium across dissonant worlds.

#### Subtheme 3.2: “I don't want to ever forget the cultural identity I come from, but there's definitely some things that I want to unlearn about it.”

This subtheme explores how migration enabled participants to re-evaluate internalised values, transitioning from cultural anchoring to ethical and cognitive transformation. Early moral codes, once taken as fixed, were revisited through the freedom to choose, reinterpret, or refuse them. Independence revealed that norms of duty and care were not universal virtues but context-bound expectations.

Independence allowed values to be reconsidered and chosen. One participant traced a shift in her own commitments: “I have a lot of different views on things that I would view differently back then, like views on marriage views on feminism… I wouldn’t even think about feminism a lot back then, but now it’s like it’s a staple in my life.” She resisted being flattened by category: “You’re putting whole countries into boxes… you need to give some freedom for the people who represent that culture to find that culture right now.” Revision was deliberate and self-directed.

Exposure to Dutch ideas of autonomy and emotional boundaries, initially perceived as distance, created reflective space. Through work, therapy, and intercultural contact, participants learned to separate moral integrity from obedience. Autonomy was redefined as self-trust rather than detachment, and freedom came to mean authorship over one’s values.

This ethical revision was not assimilation but integration: adopting practices that resonated while discarding those that constrained. Feminism, relational equality, and boundary-setting became everyday ethics rather than borrowed ideals.

Revision was not about becoming Dutch or Western, but deepening alignment with their evolving ethics. By treating belief itself as iterative, participants achieved continuity without confinement, recovering coherence by choosing, rather than inheriting, their moral worlds.

#### Subtheme 3.3: “Although I want to take care of people… I don’t want that to be because I’m a woman,”

This subtheme explores how participants enacted “boundary work”, distancing from cultural or gendered expectations that no longer aligned with their emerging self-understandings. What began as moral constraint gradually turned into ethical authorship. They learned to act without seeking external moral validation, treating independence not as rupture but as self-trust.

Boundary work meant permission to experiment and to rest. One participant embraced exploration: “try out everything… do absurd things and figure out where your thing is.” Another reset the work ethic she had inherited: “I used to look at my laptop even on Saturdays. But I think it’s sort of getting out of it. Like, OK, Hey, you shouldn’t be doing this.” Limits were reclaimed as maturity rather than failure.

Trying new experiences, making unconventional choices, or even embracing uncertainty functioned as emotional exercises in agency. Through this process, curiosity replaced caution, and self-worth decoupled from obedience.

Rest, refusal, and emotional limits were reclaimed as signs of maturity rather than selfishness. These shifts moved away from the internalised belief that self-worth must be constantly proven through sacrifice. Professional settings provided structural support: Dutch norms around work–life balance modelled alternative ethics where moderation, not overextension, defined commitment.

Across these adjustments, participants transformed care from duty into discernment. Boundary work translated internal clarity into sustainable autonomy, an embodied equilibrium between ambition, responsibility, and wellbeing.

#### Subtheme 3.4: “if they are Indian, then it’s a different way of introduction… if it’s an international person… I am a human. I am an Indian and immigrant woman after that.”

Participants described modulating self-presentation across contexts to balance visibility, connection, and authenticity. Rather than conforming, they engaged in intentional adaptation: adjusting language, gesture, and appearance to fit social expectations while preserving internal coherence. This strategic flexibility signalled belonging without erasure. External adaptation coexisted with internal freedom. Having shed moral anxiety, participants acted with greater ease: speaking openly, expressing emotion, and setting limits without fear of judgement. Language learning, especially Dutch, reinforced confidence and agency by reducing perceived distance and affirming competence.

Self-presentation was tuned to each audience. One participant managed her image at work: “I want to come off as someone who’s culturally sensitive… but also someone who says only what they know.” The same care extended to her own community: “I feel like when I’m interacting with Indians in the Netherlands… I have to sort of tread carefully to not hurt their egos.” Belonging was negotiated through constant social reading.

Reperformance functioned as relational intelligence, belonging negotiated through awareness rather than conformity. Participants learned to read contexts, shifting between humility and self-assertion to remain legible yet authentic. For some, this fluidity was empowering; for others, it exposed the ongoing labour of performing recognition. Across these accounts, identity emerged as an active, situated process curated through interaction, sustained by emotional literacy, and grounded in the desire for meaningful reciprocity.

#### Synthesis

Theme 3 reveals identity negotiation as a dynamic process balancing emotional continuity and intentional change. Participants anchored themselves in familiar cultural practices for stability amid dislocation. This grounding enabled reflective revision of inherited norms, creating boundaries that prioritized sustainability over sacrifice. Finally, reperformance allowed them to express their evolving identities in context-sensitive ways, balancing adaptation with authenticity.

### Theme 4: “I’m constantly torn between two places, and I constantly feel like there’s an increased internal conflict.”

Building on earlier themes, Theme 4 examines unresolved tensions of migrant identity. These are not failures of adaptation but reflections of structural contradictions and affective legacies. Participants experienced selfhood as being no longer who they were, yet not fully recognised as who they had become, a liminal state that embodied both transformation and the unease of partial belonging.

The central point of this theme is that adaptation left a remainder, a self that was transformed but not fully recognised. Belonging stayed partial. Self-presentation was managed by context. One participant said, “I adopt more Dutch culture when I’m in the office… it’s not on my nature. It’s just on my dressing, the way they are talking… making sure I become a part of them rather than I drag them to my culture.” Return offered no resolution. As one woman put it, “When you go back home… there are small nuances about you which make you feel that, hey, you no longer part of India anymore.” Inclusion was promised and withheld in the same moment: “They would tell me, oh, we’re a very open society… but then I’d go attend a show… and no one would sit next to me.” Structural status drew a further line, since “a lot of the government benefits… it’s not extended to a migrant.” Read through the lens of Emotional Simultaneity, these accounts show two sets of belonging held at once, neither complete. The self was no longer who it had been and not yet fully recognised as who it had become.

#### Subtheme 4.1: “I don’t feel fully Indian anymore, but I also don’t feel like I’ll ever be Dutch. It's like I'm somewhere in between…it just feels like I’m floating.”

This subtheme captures the affective cost of inhabiting two moral worlds without full recognition in either. Participants described an ongoing need to translate themselves, to explain humour, tone, or emotion, revealing a longing for effortless connection. The intimacy once taken for granted was replaced by chronic self-monitoring, where even belonging required interpretation. Returning to India exposed a different dissonance. Familiar settings demanded older performances of modesty and deference, reviving self-consciousness that migration had partially undone. Transformation thus became its own boundary: what signified growth abroad read as transgression at home.

Neither place returned a settled sense of self. Return reactivated an older self: “Even now when I go back home, I feel like I revert back to a more scared version.” The host country offered little recognition either: “…over here also, people don’t really look at you. And then that also you know, weirdly negative way how I thought about myself.” Invisibility abroad met regression at home.

Across both spaces, participants oscillated between external invisibility and internal vigilance, sustaining coherence through continual adjustment. This was not confusion but emotional simultaneity, participants learned to navigate contradiction rather than resolve it, sustaining coherence through coexistence.

#### Subtheme 4.2: “Right now it's just very difficult…I don’t feel like I belong anywhere. I’m in that phase of being a migrant.”

This subtheme highlights how participants’ internal transformation collided with a social order where inclusion was rhetorical but rarely reciprocal. Despite fluency and professional success, public openness coexisted with polite distance in everyday encounters.

Integration was demanded and unevenly returned. One participant accepted the burden of adjusting: “As an outsider, you want to make sure you can integrate… because you’re like, OK, this is how it should be like.” The promise of openness met a different reality: “They would tell me, oh, we’re a very open society… but then I’d go attend a show… and no one would sit next to me.” Inclusion stated in principle was withheld in practice.

Institutional frameworks reinforced this divide, masking inherited colonial and racial hierarchies. Rights and benefits were unevenly distributed between citizens and migrants, revealing an implicit distinction between those protected and those merely tolerated.

Integration became a moral test of responsibility, where acceptance demanded constant self-correction. Beneath adaptation lay exhaustion, the recognition that legitimacy had to be continually earned within systems structured to withhold full recognition.

#### Subtheme 4.3: “Inside the home there is a lot of sense of belonging… I do feel like I have found my little safe space.”

Within this tension, participants created small enclaves of safety in homes, friendships, and workplaces where they could exist without translation. Domestic life offered emotional reciprocity and relief from scrutiny, anchoring stability within intimate relationships.

Belonging was rebuilt in small, bounded spaces. Home offered unconditional ground: “I belong in my home with my husband.” Work offered a defined role: “My job is something that really makes me feel like OK, I have a role to play. I contribute in some way that’s important for me.” This safety was partial and class-bound. One participant caught its limit: “It’s always a bit of a reality check… you can feel assimilated here, but the reality is also I’m in a very educated bubble… the minute I step outside of that, it’s like, yeah, I’m a brown woman in a white country. Like, that’s just what it is.” Security returned in pockets rather than across the whole social field.

Work provided another bounded form of belonging, transforming labour into a space of validation. Friendships within multicultural networks brought ease and mutual understanding, allowing authenticity without vigilance. However, participants recognised that such comfort thrived within educated, cosmopolitan circles, but dissolved beyond them, where racial difference resurfaced. These spaces thus functioned less as resolution than as moments of coherence within enduring inequality.

Migrant identity emerged as continuous negotiation between inherited values and new sociocultural demands. Participants recalibrated norms yet remained provisionally recognised within dominant frameworks. Emotional simultaneity defined this condition, sustaining cultural ties while reshaping the self under unequal terms. Domestic spaces provided grounding but could not offset broader exclusions. Belonging and recognition were contingent on cultural legibility and the continual labour of making oneself intelligible, leaving coherence partial and always under negotiation.

## Discussion

This discussion draws on postcolonial, intersectional, and affective frameworks to examine how highly skilled Indian women in the Netherlands sustain moral coherence within unequal regimes of recognition. Emotional Simultaneity foregrounds wellbeing as the affective practice through which dignity endures amid contradiction.

### How do participants perceive their identity across bicultural contexts?

Participants resisted viewing identity as context-dependent performance. They grounded selfhood in enduring moral commitments such as duty, modesty, and relational care, shaped by family and community life and sedimented through caste, class, and colonial modernity.^27^ These values, emotionally anchored rather than cognitively chosen, provided moral stability even amid limited legibility in Dutch contexts.

Migration rendered these moral economies contingent. Virtues, including deference, restraint or collective obligation, were often questioned in Dutch contexts where autonomy and directness signified competence.^32^ Through Emotional Simultaneity, participants sustained attachment to both moral worlds without resolution, showing identity as affective endurance rather than strategic adjustment.

This extends acculturation and hybridity theories^19,44^ by framing adaptation as moral and emotional labour within an ethically charged third space. Building on postcolonial psychology,^35^ the study conceptualizes migrant identity within hierarchies where coherence becomes a psychosocial achievement amid competing demands for belonging and integrity.

### What challenges and opportunities do participants encounter in navigating cultural expectations and professional roles?

If identity coherence was grounded in inherited moral economies, professional life revealed how these ethics were tested within institutional hierarchies. Although Dutch institutions appeared meritocratic, they were structured by Western, masculinist, and colonial constructions that equated assertiveness, emotional restraint, and autonomy with competence. Participants’ ethics of humility and care were routinely misread as lack of ambition or independence, producing moral dissonance as inherited virtues became liabilities.

To manage this contradiction, participants lived with constant affective vigilance. Everyday interactions required anticipatory calibration: monitoring tone, expression and assertiveness to avoid being read as timid or backward. This vigilance became hidden labour, producing fatigue and self-doubt. As Fricker^45^ argues, epistemic injustice silences affective dimensions of knowing and expressing oneself when they diverge from dominant norms. The cost was psychic strain and a persistent sense of partial legitimacy.

Even inclusion-oriented arrangements, such as the parental and flexibility schemes participants described, required performing non-threatening and productivity-enhancing forms of difference. These conditions illustrate what Ghorashi and Vieten^10^ term “conditional inclusion”, where belonging depends on adopting dominant affective codes.

Within these limits, participants exercised quiet resistance. Some redefined humility as steadiness or care as reliability, preserving moral continuity while meeting professional expectations. Others relied on emotional attunement to manage intercultural interactions, using empathy and restraint as shared strengths. These acts sustained ethical persistence without directly contesting institutional power. Professional adaptation thus remained ongoing emotional labour, an ethic of survival rather than transformation within unequal systems.

### How do participants negotiate their identities in the workplace and in their personal lives?

Identity negotiation unfolded across overlapping professional, domestic and relational spheres. Ethics of care and humility shaped collaboration and leadership, while encounters with autonomy and boundary-setting fed back into family and transnational relations. Adaptation therefore occurred within a shared affective field where belonging, obligation and recognition were constantly recalibrated.

Within this field, participants negotiated legibility through measured assertion and affective control, sustaining moral coherence while meeting institutional demands. Practices developed for workplace survival, such as boundary-setting, tone management, and emotional regulation, were reapplied to caregiving and partnership roles, while inherited ethics of care continued to guide professional conduct.

Within diaspora networks, these negotiations acquired a collective dimension. Internal hierarchies reproduced dominant expectations of respectability and integration, turning adaptation into a moral benchmark. Frustration toward less-adapted compatriots revealed how external pressures were internalised, transforming negotiation into subtle boundary policing that re-inscribed inequality within the community.

Across contexts, Emotional Simultaneity captured the psychosocial work of sustaining conflicting attachments. Wellbeing was not freedom from strain but the capacity to hold it through ongoing affective regulation. Rather than harmonizing difference, participants lived it as disciplined survival that preserved dignity within persistent misrecognition. Taken together, these findings reposition identity negotiation as an ethically anchored and emotionally sustained process of adaptation under power.

### Conceptual model through cross-analysis

The Integrative Process Model of Power-Saturated Identity Negotiation conceptualized migration as a psychosocial process shaped by structural power and sustained through emotion. The findings extend this framework, showing how Emotional Simultaneity functions as both regulation and resilience within unequal conditions. Together, these refinements clarify how coherence operates, where power acts, and what dimensions shape resilience.

**Figure 2.**
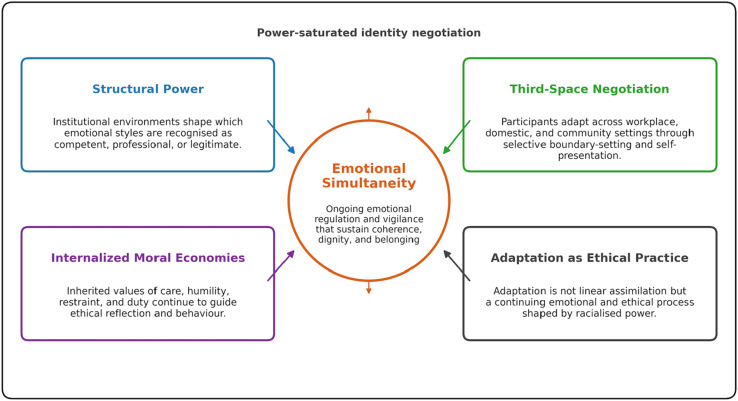
Dimensions of racialized adaptation. *Note.* This figure maps the four dimensions through which migrant women navigate racialised institutions, highlighting how power, morality, space and emotion work together to shape everyday adaptation.

As shown in Figure 2, the findings clarify how Emotional Simultaneity operates within power-saturated identity negotiation. The model brings together four interconnected dimensions: structural power, internalised moral economies, third-space negotiation, and adaptation as ethical practice. Structural Power captures how institutional environments impose racialised affective norms that regulate emotional expression and define competence. Internalised Moral Economies reflect the inherited ethical frameworks (such as humility, care and restraint) that women draw upon as guiding values. Third-Space Negotiation highlights the role of micro-arenas of safety, including homes, multicultural networks and flexible workplaces, where women can temporarily reconcile competing expectations. At the centre of these processes, Emotional Simultaneity represents the continuous emotional regulation and vigilance through which women monitor tone, visibility and expression to maintain dignity within misrecognition. Together, these dimensions show that adaptation is not a linear process but an ongoing ethical and emotional practice shaped by racialised power.

#### Emotional simultaneity as dynamic regulation

The findings refine Emotional Simultaneity from a static contradiction into a feminist-psychosocial process that sustains coherence through a continuous circulation of feeling across relational, professional, domestic, and transnational contexts. Practices of care, restraint, and composure move between these domains, showing how identity is held through responsiveness rather than resolution. This rhythm reflects Ballou and Brown’s^46^ feminist view of mental health as relational regulation within power-laden interdependence.

Within this process, coherence is not equilibrium but ethical persistence. As Huot and Rudman^13^ note, wellbeing unfolds within constraint as an ongoing negotiation of legitimacy and self-worth. Participants’ capacity to remain emotionally attuned and self-preserving aligns with Haque and Eng’s^47^ notion of reflexive vulnerability, strength as openness to contradiction without collapse.

#### Power and governance: From internalization to affective regulation

This stage reveals where power acts and moral agency emerges. While the original framework traced how internalised moral economies confront host-country institutions, the data show that this encounter is governed through feeling. Institutions regulate emotion as a condition of belonging through what Horn^48^ terms *affective colonization*, a system that rewards assertive composure, pathologizes care or restraint, and determines whose feelings count as rational or professional.

Within this regime of affective governance, participants enacted ethical reclamation. By tempering modesty without disavowing it and reframing humility as steadiness or care as reliability, they embodied what Horn^48^ calls decolonial integrity, preserving relational ethics within misrecognition. Extending this moral reclamation into the collective domain, Webb and Lahiri-Roy’s^14^ account of psychosocial resilience as socially sustained shows how women created bounded spaces of recognition where emotion was buffered and care restored coherence. What appeared as compliance thus also constituted moral authorship.

Through this lens, the model’s feedback loop is redefined from a linear exchange between internalization and institutional encounter to a continual affective negotiation of recognition. Emotional Simultaneity mediates this process: emotion operates as both the mechanism of regulation and the medium of resistance. Wellbeing thus emerges through the capacity to sustain coherence and dignity within systems that govern feeling as much as conduct.

### How emotional simultaneity operates across relation, power, and time

Building on this redefinition, the findings show that Emotional Simultaneity operates across relation, power, and time.

The relational dimension of this process is the capacity to read, anticipate, and modulate emotion within hierarchies of legitimacy, a moral intelligence rather than a behavioural skill. As feminist psychologists argue, wellbeing depends not on emotional control but on context-aware attunement within unequal relationships.^46,47^ Participants’ vigilance, knowing when to soften tone or display composure, illustrates agency as a means of sustaining connection without erasure.

A structural dimension is evident in the internal circulation of power within migrant networks. Emotional comportment becomes a measure of moral worth, how one adapts, performs confidence, or embodies respectability. These dynamics show that emotional regulation operates horizontally as well as vertically, as communities reproduce hierarchies they also attempt to resist. Yet, as Ahmed^20^ notes, emotion binds social worlds through both anxiety and attachment, turning surveillance into an ambivalent form of belonging.

A temporal dimension is also evident. Coherence is not secured once and for all but emerges in recurring moments of ease, recognition, or quiet dignity that interrupt strain. Following Cvetkovich,^49^ these fleeting synchronies form reparative time, enabling affective recovery without closure. This temporal perspective shows adaptation as cyclical rather than developmental, sustained through ongoing affective recalibration.

Taken together, these mechanisms reveal what dimensions shape resilience: relational attunement, social accountability, and temporal repair. Emotional Simultaneity thus becomes a distributed, dynamic practice of coherence sustained under inequality.

### Practical implications

As the SDG era closes, women’s mental health policy must move beyond inclusion metrics toward emotional equity. Findings call for new global frameworks that treat affective safety, dignity, and belonging as core health indicators. Health systems can operationalize these through psychosocial audits and culturally responsive wellbeing measures. Workplaces employing migrant women should build organizational awareness of how norms of assertiveness, restraint, professionalism and confidence may be racialised or gendered. This should include training and reflection for managers, HR teams, senior leaders and colleagues, so that responsibility for inclusion does not fall only on migrant women themselves. Community organisations can create collective care infrastructures that transform isolation into shared resilience. Embedding emotion as a public-health determinant^50^ reframes mental health as the foundation of sustainable adaptation beyond 2030.

### Limitations

This study examined first-generation Indian women in the Netherlands, a small, highly educated group whose socioeconomic privilege coexists with racialised and gendered marginality. Findings should be interpreted with caution given reliance on self-reported narratives, which may underrepresent collective or unconscious affective processes. We assessed quality against established criteria for qualitative validity, including sensitivity to context, commitment, rigour, transparency, and impact.^51^ Consistent with IPA, we make no claim to statistical generalisability; the findings offer theoretical transferability, and readers can judge their relevance to other settings.^39^ Future research could extend beyond migration to examine how Emotional Simultaneity operates within other forms of mobility, precarity, and systemic transition, such as climate adaptation, digital labour, and caregiving economies. Comparative and multimethod designs integrating ethnographic, physiological, and participatory approaches could generate applied frameworks for relational resilience, emotional governance, and collective wellbeing across shifting global conditions.

### Conclusion

This study shows how highly skilled Indian women in the Netherlands sustain wellbeing through Emotional Simultaneity, the ongoing affective labour of holding conflicting cultural and professional commitments. Framing wellbeing as a relational and ethical process, rather than an individual trait or a settlement outcome, extends feminist and psychosocial understandings of migrant mental health. The practical message is direct. Health, workplace, and migration systems should treat emotional safety and recognition as conditions for wellbeing, not as secondary concerns. Emotion is a determinant of health, and supporting migrant women in unequal settings should begin there.

## Supplemental Material

Supplemental Material - Navigating identity and migration: The lived experiences of Indian immigrant women in the Netherlands Supplemental Material for Navigating identity and migration: The lived experiences of Indian immigrant women in the Netherlands by Pankhuri, Sarah Jay, Gulnaz Anjum in Women’s Health.

## Data Availability

We have provided supplemental information and additional details for this paper along with the submission. Anonymized data will be provided upon request. Please reach out to the corresponding author.*
