# Supplementary material for: Navigating identity and migration: The lived experiences of Indian immigrant women in the Netherlands
Source: Womens Health (Lond). 2026 Aug 14;22:17455057261479596. doi: 10.1177/17455057261479596 (PMC13477542; doi:10.1177/17455057261479596)
Supplement: Supplementary file 1 [file sj-pdf-1-whe-10.1177_17455057261479596.pdf]

SUPPLEMENTARY MATERIAL

A. Interview Guide

Introduction

- Greet participant and thank them for their time.
  - Hello, thank you for agreeing to take part in this interview. I appreciate your time today. Before we begin, I'd like to walk you through the information sheet to make sure you have all the information about this study, so you know what to expect.
- Explain the purpose of the interview.
  - The purpose of this interview is to explore how Indian immigrant women like you navigate their sense of self and identity in a bicultural context. I am interested in hearing about your experiences and the ways in which you manage and balance your cultural identity, gender, and ethnicity as you go about your daily life and engage with various social contexts. The focus is on your personal journey of self-identity and how you navigate being both a woman and an immigrant in a new cultural environment.
- Reaffirm confidentiality and anonymity.
  - Please know that everything you share today will be kept confidential. Your personal information, including your name and contact details, will not be used in any report or publication. We will assign you a pseudonym to ensure your privacy is maintained. Any identifying information will be anonymized during the analysis process. The recording will only be used for research purposes, and your confidentiality will be protected throughout the process. The findings will be shared in my final research report, which could be published as part of my thesis or in academic journals.
  - It is important to know that your participation is completely voluntary. You are free to withdraw at any time without any consequences. If you choose to withdraw, your data will be removed from the study.
  - Do you have any questions about what is written in the information sheet or anything I've just gone over?
  - If you have any questions or concerns during or after the study, feel free to contact me or my supervisor. We are happy to address any questions or concerns you may have.
- Obtain verbal consent to proceed with the interview and audio recording.
  - Do I have your consent to proceed with this interview and the recording? If you feel comfortable, you can now give your verbal consent to participate in the study.

Part 1: Background Information

- **Can you tell me a little bit about your background?**
  - Where are you originally from?
  - Can you tell me about your family? Your childhood friends? Your school life?
- ***Part 2: Self-Identification and Introduction***
  - When meeting someone for the first time, how would you introduce yourself? What aspects of your background or identity do you mention, and why?
  - Are there cultural expectations from your family that influence how you present yourself to others?
  - What aspects of your identity do you feel are most important to you
  - Do you express your identity differently based on who you are interacting with or the context in which you find yourself?
    - do you act differently in social gatherings with people from your home country versus with people from the local dutch culture?

Part 3: Ethnic Identity in Different Contexts

  - What does 'belonging' look like to you in each context?
    - How do you manage cultural differences or similarities between your home culture and the culture of the country you live in now?

- How do you adapt your cultural practices, such as language, food, or traditions, in your everyday life here?
  - Prompt: Are there practices you continue to engage in strongly, or do you find yourself adapting them to fit in?
- How do you maintain or adapt family relationships and traditions while living in a different country?
- What role does your family play in how you navigate your identity as an Indian woman in the Netherlands?
  - In what ways do your family's cultural traditions or values shape your understanding of gender and ethnicity?
- How do societal expectations of gender and ethnicity impact your sense of self as an Indian immigrant woman?
  - (Prompt: Have you noticed any specific stereotypes or assumptions that affect how you are treated or how you view yourself?)
- Do you feel that your role as a woman in your family is different from your role as a woman in the Netherlands? What about different from that of your male counterparts?

Part 4: Sense of Belonging and Cultural Navigation

- What professional field do you work in?
- How do your family's expectations influence your career choices or professional behaviour? For example, how have familial roles such as being a daughter, wife, or mother shaped your work-life balance or career aspirations?
- In what ways do you connect with other immigrant women, particularly those from similar or different cultural backgrounds? How does this shape your understanding of who you are?
- Looking back at the person you were before moving here, how do you feel your identity has evolved? In what ways have the systems you've been a part of shaped that change?

Part 4: Wrap-up

- *Is there anything else you would like to add about how you navigate your identity across different systems?*

Part 5: Closing

- Thank you so much for sharing your experiences. Your insights are invaluable in helping to understand how immigrant women navigate their identity across multiple systems. As a reminder, everything shared here will remain confidential, and your identity will not be tied to any of the responses provided.

B. Analytic Process (Stages of IPA Analysis)

Stage 1: Idiographic Coding (Within-Case Analysis)

In keeping with IPA's idiographic commitment, analysis began with detailed, participant-by-participant coding. Each transcript was read multiple times to ensure immersion in the participant's perspective. Codes were generated line by line, capturing descriptive detail, linguistic nuance, and emergent conceptual insights. These codes were clustered within each participant's dataset to develop participant-level patterns of meaning. This approach ensured that each participant's experiential world was respected in its own terms before any cross-participant abstraction.

This section demonstrates the idiographic stage of analysis for a single participant. First, line-by-line coding was conducted and grouped into initial analytic domains (Cultural, Gender, Professional) in India and Netherlands. Each domain was analyzed separately to respect the participant's framing and meaning making. Then, all domains were considered together to identify broader superordinate experiential patterns across the participant's account. This process shows the analytic move from domain-specific coding to abstraction across the whole idiographic case.

Table B.1

Gender Domain Codes (India): Participant Aruna

Excerpt	Code
"Yeah, it could be as simple as staying out late…"	Overprotection masked as care
"I just want to do what I want to do…"	Cultural policing of self-expression

Table B.2

Cultural Domain Codes (India): Participant Aruna

Excerpt	Code
"In India, it's only family that holds me there."	Emotional anchoring through family
"India is very community based… much warmer."	Complex emotional life of Indian society

Table B.3

Professional Domain Codes (India): Participant Aruna

Excerpt	Code
"Hard working was one of the things… no matter what."	Hard work as moral imperative
"You work even Saturdays and Sundays if you have to."	Normalization of overwork

Each domain was coded separately to remain close to the participant's own structuring of their experience. This idiographic domain-level analysis ensured that cultural, gendered, and professional dimensions were respected before cross-domain abstraction.

Next, codes from all three domains were considered together to identify higher-order experiential clusters unique to the participant. These cross-domain clusters reflect how the participant integrates or negotiates different aspects of their experience.

Table B.4

Cross-Domain Clustering in India (Participant Aruna)

Codes from All Domains	Emergent Within-Case Cluster
Overprotection masked as care<br>Cultural policing of self-expression<br>Silencing of girls' voices	Gendered Surveillance and Internalised Policing
Emotional anchoring through family<br>Interrupted cultural practices<br>Emotional life of Indian society	Cultural Belonging and Emotional Attachment
Narrow professional expectations<br>Hard work as moral imperative<br>Normalization of overwork	Duty and Sacrifice as Moral Imperative

This participant's codes were first organized by domain to ensure idiographic depth within Cultural, Gender, and Professional spheres. When examined together, clear experiential clusters emerged that transcended domain boundaries, for example, gendered surveillance intersected with professional expectations of duty and sacrifice. This cross-domain clustering highlighted how moral obligations, emotional attachments, and self-surveillance were interwoven in the participant's account, informing the development of superordinate domains at the cross-case level.

Stage 2: Cross-Case Analysis for Convergence and Divergence

After idiographic participant-level coding (Stage 1), analysis proceeded to cross-case comparison within each national context. For India and the Netherlands separately, participant-level domain codes were compared and clustered into higher-order domain-level categories. This ensured shared experiential patterns within each cultural context were captured before any cross-context integration. Below, domain-level clusters for India and the Netherlands are presented for each analytic domain: Gender, Cultural, and Professional.

Gender Domain: Cross-Case Analysis

The following tables summarise domain-level clusters developed from cross-case coding within India and within the Netherlands.

Table B.5

Gender Domain Clusters (India)

Domain-Level Clusters	Description
Gender Role Expectations	Cultural production of norms; division of labour; tradition reinforcing gender roles.
Gendered Socialisation & Conditioning	Mechanisms of early learning, role modelling, emotional effects of socialisation.
Surveillance, Policing & Control	Family and society enforcing respectability, internalised self-surveillance.
Gendered Autonomy & Restriction	Mobility limits, curfews, spatial self-limitation, continuity across life stages.
Resistance, Negotiation & Identity Conflict	Critique, strategic silence, asserting agency in restricted environments.

These domain-level clusters reflect cross-case analysis within each national context, preserving cultural specificity before any cross-context comparison.

Table B.6

Gender Domain Clusters (Netherlands)

Domain-Level Clusters	Description
Negotiating Gender Roles in Relationships	Renegotiating norms in cross-cultural partnerships.
Liberation from Gendered Constraints	Opportunities to challenge and relax traditional norms.
Gendered Emotional Labour in Cross-Cultural Contexts	Continued expectation of caregiving roles even in new context.
Gendered Socialisation and Cultural Translation	Internalised norms confronted in migration, evolving over time.

Cultural Domain: Cross-case analysis within India and the Netherlands generated the following domain-level clusters.

Table B.7

Cultural Domain Clusters (India)

Domain-Level Clusters	Description
Foundational Indian Cultural Values	Moral imperatives, middle-class ethos, parental guidance.
Negotiating Bicultural Identity in Diaspora	Migration as autonomy, disidentification with norms.
Cultural Belonging and Emotional Attachment	Family anchoring, sensory-spatial belonging.
Community Surveillance and Social Judgment	Intrusion into personal life, policing of autonomy, "log kya kahenge".
Cultural Continuity Through Arts and Practices	Classical dance, religious symbolism, interrupted traditions.
Intergenerational Transmission and Transformation	Passing progressive values, retrospective appreciation.
Internalised Racism and Colonial Legacies	Colorism, idealisation of whiteness, colonial mentality critique.

Table B.8

Cultural Domain Clusters (Netherlands)

Domain-Level Clusters	Description
Belonging, Displacement, and Ambivalence	Emotional liminality, fragmented anchoring, dual cultural pull.
Selfhood, Growth, and Identity Reconstruction	Identity loss and reconstitution, personal transformation.
Structure, Power, and Systemic Barriers	Bureaucratic hurdles, racial gatekeeping, precarity.
Family, Gender, and Generational Recalibration	Shifts in kinship ties, evolving norms, intergenerational negotiations.
Cultural Strategy, Navigation, and Resistance	Deliberate preservation, selective adaptation, boundary work.
Community, Continuity, and Diaspora Life	Rituals, foodways, diaspora networks, emotional bridges.

These clusters reflect how participants in India and the Netherlands described cultural identity, belonging, and continuity, shaped by different local and migratory contexts.

Professional Domain: Cross-Case Analysis

Domain-level clusters for professional experiences were developed by comparing participant-level coding across cases within India and the Netherlands.

Table B.9

Professional Domain Clusters (India)

Domain-Level Clusters	Description
Family Influence on Career Pathways	Parental involvement, sibling modelling, gendered permissions.
Gender and Professional Identity	Constraints in education and work, double burden.
Academic Pressure and Meritocracy	Internalised performance standards, prestige-driven choices.
Bicultural Professional Identity	Tensions between upbringing and Western professional norms.
Structural Constraints and Career Redirection	Lack of guidance, trend-following, shifts in interest.
Work Culture and Well-being	Overwork, mental health impacts, toxic productivity.
Societal Expectations and Autonomy	Conformity vs. agency, cultural narratives of success.

Table B.10

Professional Domain Clusters (Netherlands)

Domain-Level Clusters	Description
Bicultural Professional Identity	Redefining self in migration, navigating differences.
Migration as Professional Reframing	Autonomy, reframing career ambitions.
Negotiating Autonomy in Work Culture	Boundary setting, new expectations.
Structural Constraints and Career Redirection	Visa issues, systemic barriers, pragmatic shifts.

This within-context cross-case analysis clarified shared pressures around family expectations and professional duty in India, and highlighted migration as a site for negotiating autonomy and reframing career trajectories in the Netherlands.

These domain-level cross-case analyses provided the foundation for later integration across contexts (Stage 3). By first examining convergence within India and within the Netherlands, the analysis preserved contextual specificity before developing final superordinate experiential clusters that could account for both convergence and divergence across settings.

Stage 3: Cross-Context Integration of Domain Analyses

After coding and clustering at the participant level (Stage 1) and developing domain-level clusters for India and the Netherlands separately (Stage 2), I conducted cross-context analysis. This stage integrated the domain-specific analyses for India and the Netherlands to identify patterns of convergence and divergence across cultural contexts, and to develop higher-order superordinate

experiential clusters. This process is detailed below for each analytic domain (Gender, Cultural, Professional).

Gender Domain: Integrating India and Netherlands Analyses

The following table compares the domain-level clusters identified in India and the Netherlands, highlighting both shared experiential structures and context-specific variations. This cross-context analysis informed the abstraction of broader superordinate clusters relevant across settings.

Table B.11

Cross-Context Comparison: Gender Domain

India Clusters (from cross-case India coding)	Netherlands Clusters (from cross-case Netherlands coding)	Shared Elements (Convergence)	Contextual Differences (Divergence)
Gender Role Expectations<br>Gendered Socialisation & Conditioning<br>Surveillance, Policing & Control<br>Gendered Autonomy & Restriction<br>Resistance, Negotiation & Identity Conflict	Negotiating Gender Roles in Relationships<br>Liberation from Gendered Constraints<br>Gendered Emotional Labour in Cross-cultural Partnerships<br>Gendered Socialization and Cultural Translation	Internalised gender norms<br>Emotional caregiving expectations<br>Policing of female autonomy	India: Norms seen as duty, enforced via family and society<br>Netherlands: More freedom to renegotiate roles, new tensions in cross-cultural relationships

This integration demonstrated shared moral expectations around gendered behaviour and emotional labour across contexts but also revealed that migration introduced opportunities for renegotiation and critique of these roles. Such convergence and divergence shaped the abstraction of superordinate clusters describing how gender norms are produced, internalised, and challenged.

Cultural Domain: Integrating India and Netherlands Analyses

Table B.12: Cross-Context Comparison: Cultural Domain

India Clusters	Netherlands Clusters	Shared Elements (Convergence)	Contextual Differences (Divergence)
Foundational Cultural Values<br>Cultural Belonging and Emotional Attachment<br>Community Surveillance and Social Judgment<br>Cultural Continuity through Arts and Practices<br>Internalised Racism and Colonial Legacies	Fragmented and Incomplete Belonging<br>Negotiated Cultural Identity<br>Rituals, Foodways and Diaspora Practices<br>Resistance to Cultural Fixity<br>Navigating Structural Racism and Gatekeeping	Importance of cultural continuity<br>Emotional anchoring in family and practices<br>Challenges of belonging	India: Stable cultural groundings but high surveillance<br>Netherlands: Fragmented belonging, adaptation of cultural practices, visible racialisation and stereotyping

Cross-context integration highlighted shared valuing of cultural continuity and belonging but also underscored the complexity of negotiating these identities in migration. The analysis informed the abstraction of clusters reflecting emotional anchoring, resistance to cultural erasure, and experiences of social judgment.

Professional Domain: Integrating India and Netherlands Analyses

Table B.13:

Cross-Context Comparison: Professional Domain

**India Clusters**	**Netherlands Clusters**	**Shared Elements (Convergence)**	**Contextual Differences (Divergence)**
Family Influence on Career Pathways<br>Academic Pressure and Meritocracy<br>Gender and Professional Identity<br>Work Culture and Well-being<br>Societal Expectations and Autonomy	Bicultural Professional Identity<br>Migration as Professional Reframing<br>Negotiating Autonomy in Work Culture<br>Structural Constraints and Career Redirection	Internalised work ethic<br>Family influence on choices<br>Moral framing of sacrifice	India: Limited pathways, overwork normalised as duty<br>Netherlands: Migration enables reframing, autonomy tensions but new opportunities

Integration across contexts showed how professional identity remained bound to expectations of sacrifice and family duty but also revealed migration as a site for reframing and negotiating autonomy. This cross-context analysis informed the development of clusters describing professional duty as moral identity and migration as an opportunity for self-definition.

This cross-context integration process was a crucial step in moving from domain-level analyses within each cultural context to identifying superordinate experiential clusters applicable across settings. These integrated insights then informed the final theme development presented in the results chapter.

Stage 4: Development of Final Themes and Subthemes

After cross-case domain-level analyses within India and the Netherlands (Stage 2), the next analytic step was to integrate these findings to develop final superordinate themes and subthemes. This involved comparing domain-level clusters across contexts to identify shared experiential patterns (convergence) and context-specific variations (divergence). Abstraction at this stage produced higher-order themes that could account for the complex, cross-cultural experiences of participants while preserving nuanced differences.

The process involved:

- Identifying which domain-level clusters from both contexts meaningfully related to each other
- Exploring how they jointly described broader experiential patterns
- Grouping these clusters into coherent, higher-order themes with theoretical and practical significance

Each final theme represents an integrated interpretation that cuts across Gender, Cultural, and Professional domains, while subthemes break down the thematic structure into specific experiential dimensions expressed in participants' narratives.

Theme 1: "I was not raised to be independent. I was really raised to be a daughter"

Stage 2 Domain-Level Clusters Feeding This Theme:

- Gender (India): Gender Role Expectations; Gendered Socialisation & Conditioning; Surveillance, Policing & Control.
- Cultural (India): Foundational Cultural Values; Community Surveillance and Social Judgment.
- Professional (India): Family Influence on Career Pathways; Academic Pressure and Meritocracy.
- Gender (Netherlands): Negotiating Gender Roles; Gendered Emotional Labour; Cultural Translation of Socialisation.
- Cultural (Netherlands): Community Surveillance and Social Judgment.
- Professional (Netherlands): Family Influence on Career Pathways.

Cross-Context Integration:

- Convergence: Participants in both contexts described internalised moral expectations shaping femininity, duty, modesty, and care as virtues.
- Divergence: India: these norms were presented as duty and virtue enforced by family and society; Netherlands: participants described these expectations as lingering internalisations they began to question and negotiate.

Analytic Rationale:
These domain clusters were grouped because they all described how participants learned to inhabit moral expectations of being dutiful daughters, caring women, and diligent students or professionals. The clusters converged on the idea of internalised moral regulation shaped by gendered, cultural, and professional expectations that were taught, policed, and sometimes challenged.

Theme 2: “Back home for someone who's 29, I'm not doing things a typical Indian 29 year old would be doing”

Short Definition:
Explores tensions between internalised moral economies and Dutch expectations of individualism, assertiveness, and directness, highlighting misrecognition, recalibration, and moral compromise in migration.

Stage 2 Domain-Level Clusters Feeding This Theme:

- Gender (India): Surveillance, Policing & Control; Gendered Autonomy & Restriction.
- Cultural (India): Community Surveillance and Social Judgment; Cultural Belonging and Emotional Attachment.
- Professional (India): Work Culture and Well-being; Societal Expectations and Autonomy.
- Gender (Netherlands): Negotiating Gender Roles; Liberation from Constraints.
- Cultural (Netherlands): Fragmented and Incomplete Belonging.
- Professional (Netherlands): Negotiating Autonomy in Work Culture; Structural Constraints.

Cross-Context Integration:

Convergence: Both contexts showed tensions between relational obligations and self-expression.

Divergence: India: norms seen as non-negotiable duties; Netherlands: migration context required recalibrating or contesting these expectations.

Analytic Rationale:
These domain clusters were combined because they described the frictions participants faced navigating different cultural moral economies. The theme captures how migration prompted participants to confront and recalibrate internalised codes of modesty, diligence, and emotional care when encountering Dutch norms of directness and individual autonomy.

Theme 3: “There are also people who move here who haven't changed a bit…”

Short Definition:
Highlights participants' active negotiation of identity through cultural retention, critical reflection, boundary work, and strategic reperformance in migration.

Stage 2 Domain-Level Clusters Feeding This Theme:

- Gender (India): Resistance, Negotiation & Identity Conflict.
- Cultural (India): Cultural Strategy, Navigation, and Resistance; Continuity through Arts and Practices.
- Professional (India): Bicultural Professional Identity.
- Gender (Netherlands): Liberation from Gendered Constraints; Negotiating Gender Roles.
- Cultural (Netherlands): Cultural Strategy and Resistance; Diaspora Practices.
- Professional (Netherlands): Migration as Professional Reframing.

Cross-Context Integration:

- Convergence: Participants in both contexts described reflecting on, adapting, and sometimes rejecting inherited norms.
- Divergence: Migration context enabled more explicit strategic adaptation and selective retention.

Analytic Rationale:
These domain clusters were grouped because they all addressed participants' intentional work to maintain cultural roots while reflecting on and revising inherited norms. The theme captures the balance between continuity and transformation in migration.

Theme 4: "I'm constantly torn between two places"

Short Definition:
Captures participants' unresolved negotiation of contradictory cultural norms and emotional simultaneity of partial belonging.

Stage 2 Domain-Level Clusters Feeding This Theme:

- Gender (India): Surveillance and Self-Regulation; Resistance and Negotiation.
- Cultural (India): Cultural Belonging and Emotional Attachment; Community Surveillance and Social Judgment.
- Professional (India): Negotiating Autonomy.
- Gender (Netherlands): Negotiating Gender Roles; Cultural Translation.
- Cultural (Netherlands): Fragmented and Incomplete Belonging; Emotional Anchoring in Diaspora Life.
- Professional (Netherlands): Bicultural Professional Identity.

Cross-Context Integration:

- Convergence: Persistent ambivalence and emotional work of maintaining ties to multiple cultural logics.
- Divergence: Different ways partial belonging and suspended identity played out depending on context and migration trajectory.

Analytic Rationale:
These clusters were grouped because they all described the ongoing emotional labour of navigating contradictory attachments and expectations without fully resolving them. The theme highlights the experience of partial belonging and suspended identity. This appendix section documents the analytic transition from detailed idiographic coding (Stage 1), through cross-case domain-level clustering within India and the Netherlands (Stage 2), to cross-context integration and abstraction into final themes and subthemes (Stage 3).

C. Final Themes and Subthemes

Summary Table of Final Themes and Subthemes

Grand Master Thematic Table

Theme	Subtheme	Short Definition
1. “I was not raised to be independent. I was really raised to be a daughter”	1.1 “They always wanted you to be soft. The good girl”	Gendered socialisation promoting passivity, gentleness, moral respectability, and suppression of assertiveness as essential to femininity.
	1.2 “They would always tell us to be humble and modest”	Moral instruction in humility, modesty, and deference, enforced through familial and social expectations shaping women's conduct.
	1.3 “I always paid attention to studies… so I do not need to bring my parents down”	Academic diligence and professional aspiration framed as moral repayment for familial sacrifice and a source of respectability.
2. “Back home for someone who's 29, I'm not doing things a typical Indian 29 year old would be doing”	2.1 “I was just taking it quietly… because that’s how you are raised”	Clashes between internalised deference and Dutch expectations of directness, where respectful silence was misinterpreted as passivity.
	2.2 “The nightmare that your Indian work life is… that trauma has just come to you in this new country”	Ethic of overperformance rooted in moral obligation, clashing with Dutch work norms and producing dissonance over balance and self-worth.
	2.3 “Here generations don't interact…”	Cultural dissonance in emotional intimacy and familial connection, as collectivist expectations of care and intergenerational bonds meet Dutch individualism.
3. “There are also people who move here who haven't changed a bit…”	3.1 “It’s always gonna be India...”	Conscious preservation of cultural practices and values as emotional anchors that sustain identity and maintain ties with family.
	3.2 “I don't want to ever forget the cultural identity I come from, but there's definitely some things I want to unlearn”	Critical reflection on inherited cultural and gendered norms, with intentional efforts to unlearn restrictive aspects.
	3.3 “Although I want to take care of people… I don't want that to be because I'm a woman”	Boundary work resisting gendered expectations of self-sacrifice, redefining care as intentional rather than obligatory.

	3.4 “If they are Indian, then it’s a different way of introduction…”	Strategic adaptation of self-presentation across contexts to navigate bicultural expectations without compromising authenticity.
4. “I'm constantly torn between two places”	4.1 “I don't feel fully Indian anymore, but I also don’t feel like I’ll ever be Dutch”	Captures identity suspension and emotional simultaneity as participants navigate cultural in-betweenness, belonging nowhere and to both places at once.
	4.2 “I think right now it's just very difficult 'cause, I don't feel like I belong anywhere”	Highlights emotional strain of partial belonging, social misrecognition, and conditional inclusion despite adaptation.
	4.3 “Inside the home there is a lot of sense of belonging”	Describes domestic and intimate spaces as partial refuges offering affective anchoring amid public exclusions.

D. Supporting Quotes by Theme

Theme 1: "I was not raised to be independent. I was really raised to be a daughter"

Table C.1

Subtheme	Short Definition	Additional Supporting Quotes
1.1 "They always wanted you to be soft. The good girl"	Gendered socialisation promoting passivity, gentleness, moral respectability, and suppression of assertiveness as essential to femininity.	"You couldn't always voice your opinion or you couldn't always say things… Make sure that your opinions were kept to yourself."
		"Some people constantly kept telling you that no, you should not do that, because what will the boys think of you? You should not sit a certain way."
		"You were always cautious about what you wore around people… you always had to cover everything."
		Interestingly, I think it's a very conditioning thing in a Indian female, which is so much ingrained in our DNA
		So it was something as simple as what I was allowed.. to wear or what I was allowed to eat…, how you behave, how you talk to elders, how you sit in front of them.
		I would still not be able to wear like crop tops and stuff because it's just something that is instilled in you to look a certain way to dress a certain way
		"I think there is an underlying assumption that as a woman, you have to really do more than what an average man would have to do."
		"Everything was in such a way that all the restrictions were always on the girls."
		"We never really had the chance to express ourselves, even with uniforms, they controlled how we looked."
		"My parents were very protective of me… so I didn't always get to do some stuff like staying out late or going alone."
		there is a lot of also increased obligation, if you are the only daughter. So I am the only daughter. So the kind of emotional toll and obligation that you have is much higher compared to your brother's. There's a sense of freedom that they kind of take for me
1.2 "They would always tell us to be humble and modest"	Moral instruction in humility, modesty, and deference, enforced through familial, educational, and social expectations that shaped women's conduct and cultural identity.	"We, we grew up in sort of army quarters throughout my upbringing… I think there is always a lot of values associated with that and you know you should remember where you come from. But you should work really hard, so I think those things get really ingrained in you and you should be respectful."
		"My both my parents are very generous people… they would do it without much

		expectation… I've seen my dad treat people in the service industry very well… that's something my brother and me also follow even now.”
		I think it was mostly Indian society as a whole that shapes you
		I did notice that like while growing up even even from age of like 10 or 12 people would would be very OK making comments about like my my body or or how I look
		“In India, everyone tries to soften their blow. Whatever they're saying, try to be really nice, even if they're saying something that wouldn't be so nice to hear.”
		you couldn't just go out on the street and just be yourself. There were always people saying things. People just looking at you weird.
		But there I feel perceived. I feel like I sound I like. There are eyes on me which is which is an uncomfortable and a very unsafe feeling. And I think I lose a sense of independence when I go to go to India because again, it's it's the the safety aspect comes into play
1.3 “I always paid attention to studies… so I do not need to bring my parents down”	Academic diligence and professional aspiration framed as moral repayment for familial sacrifice and a source of respectability, often gendered as duty.	“the effect of both of it, that was that they both sort of made sure that like we both studied really well. So our our extracurricular activities were on the side and it was always like oh, like you need to focus on academics a lot.”
		my older brother.<br>They pushed him to be an engineer, but he did liberal arts in the end and for me it was almost like they were quite unhappy about it. I was very technical, like it almost felt like why? Like it was for me, for my education. It was a huge financial argument
		While I still see men in India, of course it's better than, let's say, my parents generation, but I still see that even as professionals, as women who are working at the same level as men, getting paid the same amount, they still have the added burden of doing more when when they're in India just because of their setting
		“It was a mix of both because there was my parents did not really put as much pressure, but it was also like… if you did well in the previous grade, then you should try to continue to do well and not dip. I just thought that that was the only way to go forward.”

		"I think there is a lot of internal drive because externally… my brother is not like me at all… For me, that was just never enough and I really don't remember a time when I didn't feel that way… I always wanted to do… be the best."

Table C.2

Theme 2: "Back home for someone who's 29, I'm not doing things a typical Indian 29-year-old would be doing"

Subtheme	Short Definition	Additional Supporting Quotes
2.1 "I was just taking it quietly… because that's how you are raised"	Clashes between internalised deference and Dutch expectations of directness, where respectful silence was misinterpreted as passivity or incompetence.	"I don't speak up or... become very non-confrontational with them... I carry then a lot of resentment sometimes."
		"I can't seem to bring myself to... say hey I did not like that... maybe because I've been raised to like, yeah, you know, respect the elders."
		"If you speak good English, it's always quite shocking... assumption becomes that you're not from where you're from."
		"Something was stopping me from saying it... sometimes I still have to think like, is it OK? Is it not OK?"
		"I would have wanted to say something, but... I go back and think about it and I'm just like, no, no, no, that's not OK."
2.2 "The nightmare that your Indian work life is… that trauma has just come to you in this new country"	Ethic of overperformance rooted in moral obligation, clashing with Dutch work norms and producing dissonance over balance, guilt, and self-worth.	"I would hate myself if I ever thought I was being complacent... yeah, it is difficult. You cannot force here that in a professional environment."
		"Here... if you do extra, you're not gonna get anything better... Also it spoils the work."
		"So I work more than others because again, that's a very Indian thing that you work hard, you work hard to succeed."
		"I just kind of keep pushing myself... personally I want to do more... try to be more forceful towards myself there."
		"Always a push and pull... I'll do like overwork. I'll do like extra hours."
		"Hectic, manic productivity time will happen for like a month... and it will burn out."
		"I wanted to dig more... find out why... but they were just like, it's not needed."
		"I'm get restless again because I'm like I'm not doing enough at my time. I feel like I'm not being productive."
	Cultural dissonance in emotional intimacy and	"Here in Netherlands, it's quite individualistic... That was quite a shock."

2.3 “Here generations don't interact…”	familial connection, as collectivist expectations of care and intergenerational bonds met Dutch individualism.	“In India where people can drop in any time... here... even to have a cup of coffee, you have to have a appointment a week before.”
		“If I said… my family needed something from me… they’d say, but it’s your family’s thing to do.”
		“With the Dutch people, it's a little more effort... more formal.”
		“It’s socially a bit draining for me to be with Dutch people... they’re OK with that... small talk... I don’t like small talk at all.”
		“Here it’s a lot more formal... I wanna just, like, go and, like, hug everyone and... joke... but here... not.”

Table C.3

Theme 3: “There are also people who move here who haven't changed a bit…”

Subtheme	Short Definition	Additional Supporting Quotes
3.1 “It’s always gonna be India...”	Conscious preservation of cultural practices and values as emotional anchors that sustain identity and maintain ties with family.	“I do celebrate a few things once in a while with some very close friends here, but then that's not just Indian friends. We also invite our Dutch friends over.”
		“Something as simple as if you're sitting on a dinner table with a lot of people you let someone else eat first.”
		“So here we do have a large Indian community…I speak Malayalam that ymy native language… I feel like that's my most comfortable place”
		“Now I've really started cooking my own food and got all my recipes from my grandparents and mother and I'm like enjoying my cooking. I used to miss home and I think now I'm less missing home”
		“We do a little bit of potluck here and there... we would put the floral pattern on the floor here.”
		“Values something as simple as treat the guest like God. So I am an amazing host, no matter who comes at my home.”
		“Of course I have calls with my family back home like I think we call almost every day.”
3.2 “I don't want to ever forget the cultural identity I come from, but there's definitely some things I want to unlearn”	Critical reflection on inherited cultural and gendered norms, with intentional efforts to unlearn aspects seen as restrictive or outdated.	“You're putting whole countries into boxes… you need to give some freedom for the people who represent that culture to find that culture right now.”
		“I have a lot of different views on things that I would view differently back then, like views on marriage views on feminism… I wouldn't even think about feminism a lot back then, but now it's like it's a staple in my life.”

		“I think that's the unfortunate part of trying to enforce values too hard... you really just resent it.”
		“I think it just helps to observe people in their natural habitat at work when they speak about their families. Ok like this not how you do it here and that's very refreshing.”
		“a very changed perspective in the last five years that I've moved here, I don't look at cooking as a gendered labour, which I used to while while growing up”
		“you do feel very like, like, what the hell, like, have I been living in this for that many years and like not realise that this was just weird, that we have to do everything.”
		“Initially I would just be like let it be... now I try and somehow always go back... tell them... this is not what should have said.”
		“I realized that I kind of broke out of all of that and I had this new life altogether and I could just like spread my wings and do whatever I feel like.”
		“you were like a golden fish in a bowl at house and then I was thrown into an ocean.”
3.3 “Although I want to take care of people… I don't want that to be because I'm a woman”	Boundary work that resists gendered expectations of self-sacrifice, redefining care as intentional rather than obligatory.	“I used to look at my laptop even on Saturdays. But I think it's sort of getting out of it. Like, OK, Hey, you shouldn't be doing this.”
		“So I would say in the past six months... I went through that hectic like oh, I want to prove myself thing, but then I had to course correct.”
		“I think you feel more empowered as a woman over here because you can just be confident and do what you want to do so that that I think is really good.”
3.4 “If they are Indian, then it’s a different way of introduction…”	Strategic adaptation of self-presentation across contexts to navigate bicultural expectations without compromising authenticity.	“I also think it helps with my confidence here because I can clearly tell them what I can and cannot do and they would not judge me for that.”
		“Learning the language really helps... because for them English is more like a professional language, but Dutch is more like, oh, I'm just making conversation with you.”
		“I will always present an Indian meal… but my home is very Western.”
		“If we show some sort of inclination towards their culture and language and food... that also gets some brownie points.”

		"I feel like when I'm interacting with Indians in the Netherlands… I have to sort of tread carefully to not hurt their egos."

Table C.4

Theme 4: "I'm constantly torn between two places"

Subtheme	Short Definition	Additional Supporting Quotes
4.1 "I don't feel fully Indian anymore, but I also don't feel like I'll ever be Dutch"	Captures identity suspension and emotional simultaneity as participants navigate cultural in-betweenness, belonging nowhere and to both places at once.	"Like the more years pass by and I visit India... the sense of belonging is a very strange feeling in both places actually."
		"Even now when I go back home, I feel like I revert back to a more scared version"
		"It's too much now for me, going home… people commenting on your weight… I want to do my own sheets and stuff… they never bothered me until I lived here."
		"Like the more years pass by and I visit India... the sense of belonging is a very strange feeling in both places actually."
		"...over here also, people don't really look at you. And then that also you know, weirdly negative way how I thought about myself."
		"I think sometimes I do find myself taking a lot of responsibility around the house and I don't necessarily have to because I have a partner who's more than willing to"
		"Like now, my marriage works the way it works here and the the way it works there, it's very different…I don't feel my gender here. But in India, I'm very much a reminder of the fact that I'm a woman and like he's a man like my husband"
		"It's been almost 17 years since I've left home. I'm in one of the most safest countries in the world, but the other day I was by myself…I was like jogging and there were like two men who walked in front of me.<br>There were two Dutch men.<br>I suddenly had this fear that Oh my God, there's no one around me. And when we pass them, they nicely, you know, smiled at me."
		"I've become more temperamental. I think because I'm constantly torn between the two places, like all the time."
		"I have spent seven years. I still have made that effort... but there is nothing that will keep me here... I would rarely be heartbroken if I have to leave."

4.2 “I think right now it's just very difficult 'cause, I don't feel like I belong anywhere”	Highlights emotional strain of partial belonging, social misrecognition, and conditional inclusion despite adaptation.	“Sometimes I feel like I'm not that Dutch enough yet… maybe that could be like a obstructing factor.”
		“If you come in from India… you start in as a fresher over here… they argue… you don’t have the relevant work experience.”
		“We were all in the assumption that the Netherlands would be very forthcoming to internationals… but it wasn't that easy.”
		“They see you as a tiny woman... who doesn't know anything.”
		“The thought process... Bollywood... Taj Mahal... snake charmers... a lot of poverty.”
		“I walked in behind someone who's another Indian woman... and he was like, oh, are you guys sisters?... they don't ever say it to other Dutch women.”
		“So sometimes they're curious. On most occasions in a positive way but also on a lot of occasions they'll ask a lot of like weird questions. I guess it's like because they don't know any better, so things like are there cows everywhere on the streets? do you get ice cream in India? is it like really poor over there isn't like 1 euro like ₹500 or something which is so far from the truth”
		“But I think just for my skin tone and colour there's so many times that I'm the only brown person in the room or in this town when I visit”
		“I think you will always have a bit more frustrations because you have to figure out the whole system as a woman and some people who are born here probably don’t have to navigate that.”
		“a lot of the government benefits that you get as being a new parent is not extended to a migrant…that level of being like If I'm not working, at least the government might support me a bit so I can take a step back now and focus on one thing. But then as migrants, you just don't have that option. You have to make a choice. It's either one or the other”
		“I feel like if I do decide to have kids, my career will take a hit... even if the company will say... You can come back.”
		“Do I feel like I belong here after seven years? Yeah... things are not pushed down on you. You can be who you want to be in that sense and people accept you.”
		“You really realise how disadvantaged you are when you don't have a European passport.You are focusing on the disadvantages caused by your culture and your heritage…I also have

		friends who kind of completely let go of it. Like whatever, I'm just Dutch now.”
		“Honestly, I don't feel I still am 100% into it or I feel that I belong to this place though. Yes, with every passing day that comfort level increases.”
		“I also think that as you grow up, this answer changes because the idea of belongingness changes.”
		“If we have kids, the holiday will be going to India... I don't care because it's important to go home and make sure my kids would know where I'm from.”
4.3 “Inside the home there is a lot of sense of belonging”	Describes domestic and intimate spaces as partial refuges offering affective anchoring amid public exclusions.	“My job is something that really makes me feel like OK, I have a role to play. I contribute in some way that's important for me.”
		“Here, my friends, my community makes me feel at home, quite like knowing I can just, you know, be like, hey, man, help me move a table. Like that kind of comfort is there.”
		“It's always a bit of a reality check. Like, you know, 'cause, you can feel assimilated here, but the reality is also I'm in a very educated bubble. I'm surrounded by people who are typically well-traveled, well-exposed, and educated, and not everybody is like that. So the minute I step outside of that, it's like, yeah, I'm a brown woman in a white country. Like, that's just what it is.”
		“I feel very much at home when I’m in my apartment over here with my fiancé… I don’t think I could have done it or come this far in the Netherlands without him."

E. Reflexivity Statement

As researchers engaged in critical qualitative inquiry, we recognize that our own positionality has shaped the interpretation offered in this discussion. Especially the first and third authors' academic and personal background sensitizes us to issues of power, postcolonial legacies, and gendered constraints, which inevitably foreground certain analytical lenses over others. While this perspective enables a structural critique attentive to epistemic injustice and institutional misrecognition, it also risks underplaying participants' own capacities for agency, reinvention, and diverse meaning-making.

We are aware of the potential for over-identification with participants' experiences of marginalization, which can lead to interpretations that stress constraint and compromise while insufficiently recognizing moments of autonomy, transformation, and dissent. In seeking to illuminate the ways institutional contexts shape adaptation as a morally charged negotiation, we may have privileged coherence in the analytical narrative at the expense of fully embracing the complex, contested, and sometimes contradictory character of participants' accounts.

This analysis also reflects our interpretive choices as authors about which themes to highlight and how to situate them within existing theoretical frameworks. While the Integrative Process Model of Power-Saturated Identity Negotiation offers valuable insights, we do acknowledge that no single model can exhaustively represent the complexity of migrant identity work across varied contexts, histories, and personal trajectories. Therefore, throughout this project we have done our best to maintain critical reflexivity about the partial and situated nature of this account, recognizing both its contributions and its limits. This reflexive stance does not undermine the analysis but situates it as one interpretive reading among many possible, open to challenge, refinement, and enrichment through ongoing dialogue with participants, scholars, and practitioners committed to understanding the layered realities of migration and belonging.
